# Supplementary material for: Healthcare providers’ perceived support from their organization is associated with lower burnout and anxiety amid the COVID-19 pandemic
Source: PLoS One. 2021 Nov 19;16(11):e0259858. doi: 10.1371/journal.pone.0259858 (PMC8604356; doi:10.1371/journal.pone.0259858)
Supplement: S1 Appendix — (DOCX) [file pone.0259858.s001.docx]

**S1 Appendix: Recruitment E-mail**

Dear Healthcare Provider,

COVID-19 has intensified the stress that many of us already experience as healthcare professionals. **We are requesting your participation in a survey-based study that explores anxiety and burnout.**We view this study as a critical first step in addressing how the COVID-19 pandemic impacts the mental well-being of front-line providers.  **Participants who complete this study will be given a random chance to receive a $250 VISA giftcard (2 available) or $20 Starbucks giftcard (25 available).**Selection will be made upon completion of the study and incentive will be distributed within 2 weeks of selection.

If you agree to participate your survey data will be deidentified so that your personal information will remain completely anonymous throughout the entire duration of the study.

The entire study will take approximately 7 months to complete with 6 monthly surveys administered. Some participants may be asked to watch a 1 hour video produced by the NIH that discusses practical and easy strategies for alleviating stress related to this pandemic. You can expect that, in total, you will spend 1-2 hours of your time on study-related tasks.

In this difficult time, we expect that many are experiencing anxiety surrounding caring for patients. Our survey is validated for use in assessing anxiety, support, and burnout. While we do not anticipate that our study will cause additional anxiety or stress for the participant, there is a risk that these may occur for some. While there are no direct benefits to the participant for completing the survey portion of this study, individuals who watch the NIH video may develop coping skills that allow them to deal with anxiety and stress, and potentially minimize their risk for burnout.  Participation in this study is entirely voluntary. You may withdraw from this study at any point. If you choose to withdraw from this study, all contact and data collection will immediately cease, and any data your have already provided will be deleted upon request.

If you are interested in participating in this study, please review the attached consent form carefully and click the following link to complete the **pre-study survey** (after which you will be sent a link for Survey 1):

https://www.ctsiredcap.pitt.edu/redcap/surveys/?s=FXYXFKW9NN

Thank you in advance for your help with this study!
