# Supplementary material for: Healthcare providers’ perceived support from their organization is associated with lower burnout and anxiety amid the COVID-19 pandemic
Source: PLoS One. 2021 Nov 19;16(11):e0259858. doi: 10.1371/journal.pone.0259858 (PMC8604356; doi:10.1371/journal.pone.0259858)
Supplement: S2 Appendix — (DOCX) [file pone.0259858.s002.docx]

**S2 Appendix: Consent form**

**Study Summary**

***Exploring provider burnout during the COVID-19 pandemic***

You are being asked to join a research study. You do not have to join the study. Even if you decide to join now, you can change your mind later.

1. The purpose of this study is to learn more about how the COVID-19 pandemic is affecting the frontline healthcare workers. We hope to understand the relationship between anxiety and burn out for those who are caring for patients throughout the pandemic.
2. In this study, we will collect information about you, your level of anxiety and burn out, as well as the support that UPMC is providing you during this time.
3. All participants will be asked to complete a deidentified survey every month for 6 months. This survey will take you less than ten minutes to complete.
4. Some participants will also be asked to review an online module, which will take about one hour, that deals with anxiety and stress during a difficult time. We anticipate approximately 250 individuals will choose to participate in this study, and that 20% of these individuals will review the online module.
5. There is no in person follow up required.
6. Please see pages 3-4 to review all possible risks.


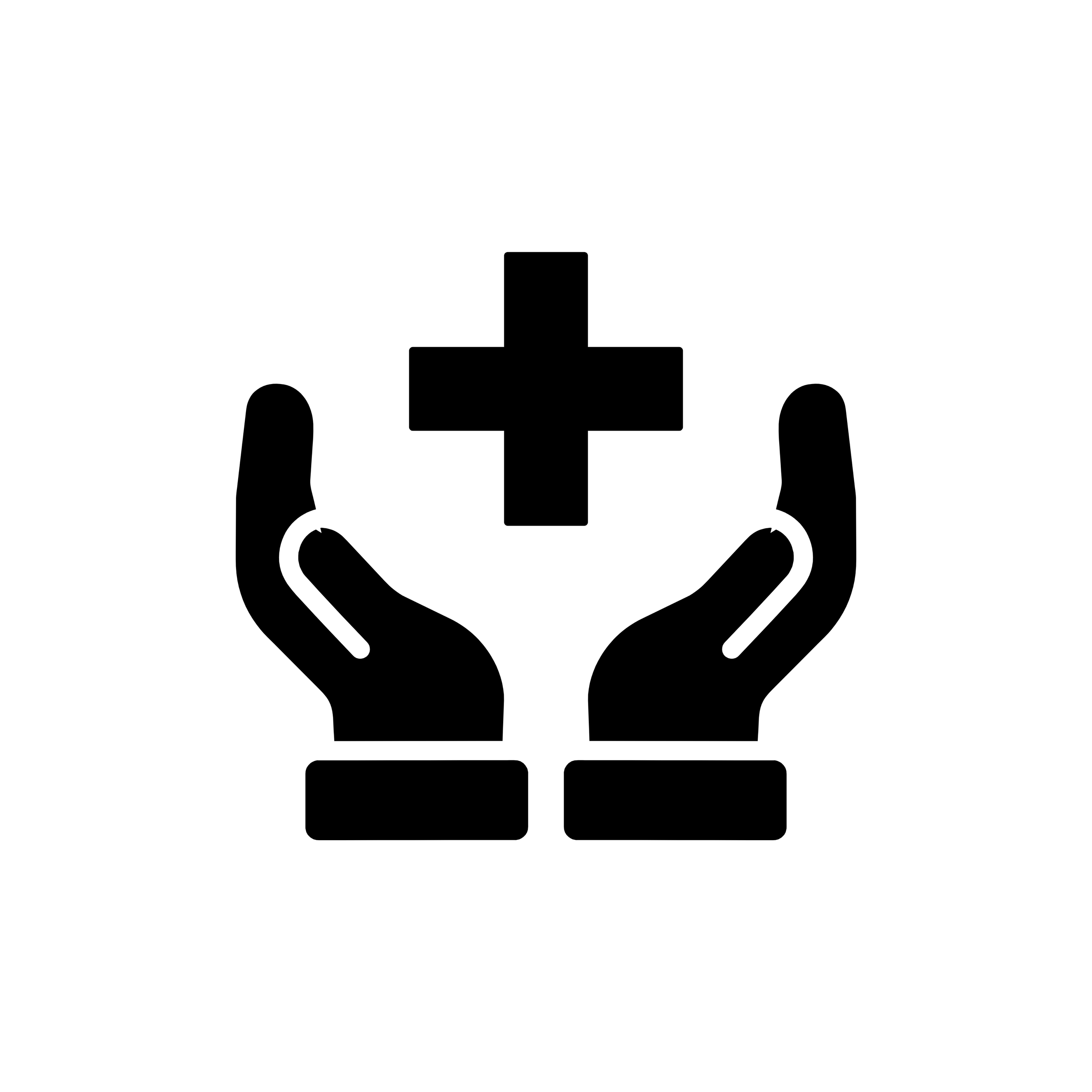


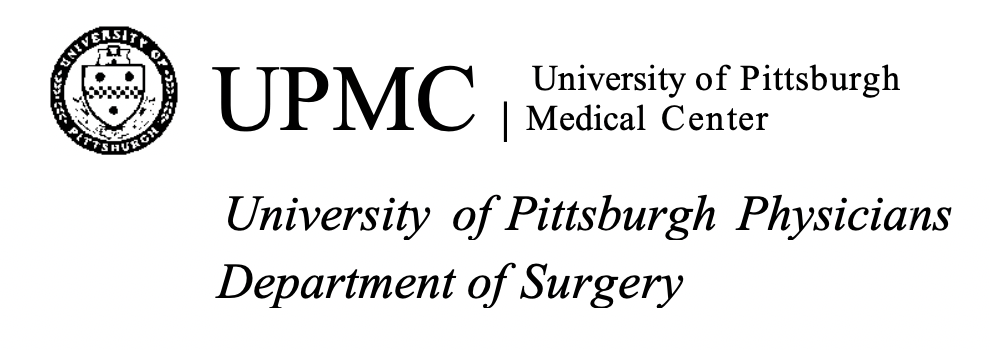


**HIPAA AUTHORIZATION AND CONSENT TO ACT AS A PARTICIPANT**

**IN A RESEARCH STUDY**

**TITLE:** Exploring Provider Burnout during the COVID-19 Pandemic

**PRINCIPAL INVESTIGATOR:** Sara P. Myers, MD, PhD

Department of Surgery

University of Pittsburgh Medical Center

Email: [myerssp@upmc.edu](mailto:myerssp@upmc.edu)

**CO-INVESTIGATORS:** Katherine M. Reitz, MD MSc

Matthew R. Rosengart, MD MPH

Emilia Diego, MD

Galen Switzer, PhD

***Why is this research being done?***

The purpose of this research study is to understand the emotional response that providers have to the COVID-19 pandemic. Specifically, we are interested in the relationship between anxiety, burn out, and the perceived support provided to healthcare workers. Burn out has been linked to both poor provider and patient outcomes and therefore is especially important at this time.

***Who is being asked to take part in this research study?***

We are asking all UPMC employees who have face to face interactions with patients at UPMC hospitals to participate in this study. Employees from the following UPMC sites will be included: Presbyterian/Montefiore, Shadyside, Altoona, Bedford, Chautauqua, Children’s Hospital, Cole, East, Hamot, Horizon, Jameson, Kane, Magee, McKeesport, Mercy, Northwest, Passavant, Pinnacle, Somerset, St. Margaret, Susquehanna, and Western Psychiatric.

***What procedures will be performed for research purposes?***

To participate in this study, you will be asked to read and sign this informed consent form before you are enrolled in this study.

Via an online survey, we will ask you questions about who you are, your stress levels, and the support that you are receiving throughout the COVID-19 pandemic. We will ask you to complete this survey a total of six times. The surveys will be collected once per month. Therefore, the entire study is expected to take less than 7 months in total.

**Survey 1**: All participants will complete this survey. This will include questions about you (<2 minutes) and a <30 question survey (5 minute).

**Intervention**: Approximately 20% of participants will be randomly selected to complete a 1-hour online intervention. These participants will be asked to participate in a 1-hour NIH-led seminar discussing 9 practical strategies for coping with crisis-related stress.

**Survey 2-5**: For all participants. This will include a <30 question survey (5 minute).

***What are the possible risks, side effects, and discomforts of this research study?***

Risks associated with confidentiality: A breach of confidentiality is a possible risk due to the fact that the study team will give you a link to complete a survey in for REDCap (the survey website). Survey data will be deidentified but initially linked using your email. This allows us to monitor your surveys overtime without you having to put any identifiable information into the survey itself. However, the study team will have a linker record ID which associates the survey to your email address. For this reason, additional protections will be taken with the information linking the codes with your identity (i.e., your name or other information that people could use to learn that you participated in this study). The linking code and your identity will be kept on a protected computer server. Only the research team involved with the study will have access to this information. There will be no paper records maintained.

All electronic records that could identify your involvement will be stored in password-protected files. These locked/protected files will contain both your name and a code that will be substituted for your name on all other files and data gathered in this study. All subjects will be provided with a recordID that will link any/all patient identified data (stored securely and protected on the UPMC/University of Pittsburgh protected patient server) with their de-identified data. Although it is highly unlikely, there is still the possibility that information on your identity could be linked back to your research information.

Email and phone may be used to contact patients to remind them of their participation in the study. No private information will be provided by the research team.

Notably, the individuals answers that each participant provides to this survey will NOT be provided to UPMC or their employers.

Risks associated anxiety: In this difficult time, we expect that many participants are experiencing anxiety surrounding caring for patients in the COVID-19 pandemic. Our survey tools are validated for their use in assessing anxiety, support, and burnout and therefore we do not expect them to cause additional anxiety or stress for the participant. However, if this is the case the participant will be free to contact the principal investigator who can help guide the participant to either mental health or medical care, as appropriate.

***What are possible benefits from taking part in this study?***

Those participants asked to watch a 1 hour long video produced by the NIH that discusses practical and easy strategies for alleviating stress related to this pandemic

(i.e., participants randomized to the treatment arm) may develop coping skills that allow them to deal with their anxiety and stress in order to minimize their burn out. Society at large might benefit from the results of this study.

***If I agree to take part in this research study, will I be told of any new risks that may be found during the study?***

You will be promptly notified if any new information develops during the conduct of this research study which may cause you to change your mind about continuing to participate.

***Will I be paid if I take part in this research study?***

No, you will not be paid.

***Will this study involve the use or disclosure of my identifiable medical information?***

This research will not review or involve the generation of information that will be placed within your medical record. Your research data may be shared with investigators conducting similar research, however, this information will be shared in a de-identified manner.

***Who will have access to identifiable information related to my participation in this study?***

In addition to the University of Pittsburgh and UPMC investigators, listed on the first page of this authorization (consent) form and their research staff, the following individuals will or may have access to identifiable information related to your participation in this research study:

Authorized representatives of the University of Pittsburgh’s Research Conduct and Compliance Office and other groups or organizations that have a role in this study will have access to and may inspect research records due to your participation in this study. This access is necessary to ensure the accuracy of the findings and your safety and welfare. If any publication or presentations result from this study, you will not be identified by name. Results will be reported in a such a way that you cannot be identified.

We will protect the privacy and confidentiality of your records, as described in this document, but cannot guarantee the confidentiality of your research records, including information obtained from the survey, once your personal information is disclosed to others outside UPMC or the University.

We may use emails to send you study information and reminders.

We will do everything possible to protect your privacy and confidentiality, but information transmitted over the internet is insecure and no method of electronic storage is perfectly secure therefore absolute confidentiality cannot be guaranteed.

***Is my participation in this study voluntary?***

Your participation in this research study is completely voluntary. (Note, however, that if you do not provide your consent for the use and disclosure of your information for the purposes described above, you will not be allowed, in general, to participate in the research study.)

***May I withdraw, at a future date, my consent for participation in this study?***

You may withdraw, at any time, your consent for participation in this research study, to include the use of your survey information for the purposes described above. Any research information recorded for, or resulting from, your participation in this research study prior to the date that you formally withdrew your consent may continue to be used and disclosed by the investigators for the purposes described above.

To formally withdraw your consent for participation in this research study you should provide a written and dated notice of this decision to the principal investigator of this research study at the address listed on the first page of this form.

Your decision to participate in this study or withdraw your consent for participation in this research study will have no effect on your current or future relationship with the University of Pittsburgh. Your decision to withdraw your consent for participation in this research study will have no effect on your current or future medical care at a UPMC hospital or affiliated health care provider or your current or future relationship with a health care insurance provider.

***If I agree to participate in this research study, can I be withdrawn from the study without my consent?***

Your participation in this study may be discontinued without your consent for the following reasons:

o if study procedures appear to be harmful to you

o if you fail to follow directions for participation in the study

o if it is discovered you do not meet the eligibility requirements

o if the study is canceled

**VOLUNTARY CONSENT**

All the above has been explained to me and all of my current questions have been answered. I understand that I am encouraged to ask questions about any aspect of my participation in the research study at any time, and that such future questions will be answered by the investigators associated with the study or their research staffs. I understand that a copy of this consent form will be given to me.

I understand that any questions, which I have about my rights as a participant in the research study, will be answered by the Human Subject Protections Advocate of the IRB Office, University of Pittsburgh

(1-866-212-2668).

**Please indicate that you provide consent to participate in this study by clicking “I agree,” in response to the following prompt on our initial survey: “I have reviewed the consent form and agree to participate in this study.”**
