# Supplementary material for: Healthcare providers’ perceived support from their organization is associated with lower burnout and anxiety amid the COVID-19 pandemic
Source: PLoS One. 2021 Nov 19;16(11):e0259858. doi: 10.1371/journal.pone.0259858 (PMC8604356; doi:10.1371/journal.pone.0259858)
Supplement: S3 Appendix — (DOCX) [file pone.0259858.s003.docx]

**S3 Appendix: Enrollment (baseline) survey**

| **Item** | **Response categories** |
| --- | --- |
| I agree to participate in this study. | - I agree |
| Please indicate your preferred email address at which to receive study-related communications. |  |
| What is your job title at UPMC? | - Attending physician - Resident physician or fellow (i.e., clinical trainee) - Advanced practice provider (e.g., Nurse Practitioner, Physician’s Assistant, etc.) - Nursing staff - Other staff (i.e., Respiratory Therapist, Patient Care Technician) |
| At which UPMC hospital do you spend the majority of your time working? | - Presbyterian/Montefiore - Shadyside - Altoona - Bedford - Chautaqua - Children’s Hospital - Cole - East - Hamot - Horizon - Jameson - Kane - Magee - McKeesport - Mercy - Northwest - Passavant - Pinnacle - Somerset - St. Margaret - Susquehanna - Western Psych - Other |
| Please indicate whether you work at an academic or community site based on the hospital at which you spend the majority of your time. | - Academic (Presbyterian/Montefiore, Shadyside, Children’s hospital, Magee, Western Psych) - Community (Altoona, Bedford, Chautauqua, Cole, East, Hamot, Horizon, Jameson, Kane, McKeesport, Mercy, Northwest, Passavant, Pinnacle, Somerset, St. Margaret, Susequehanna, Other) |
| What is your age? | - 24 years of age or under - 25-44 years of age - 45-64 years of age - 65 years of age or older - Decline to answer |
| What is your sex? | - Male - Female - Other - Decline to answer |
| What is your race? | - White, European or Middle Eastern - Black - Asian - Native Hawaiian or Other Pacific Islander - Other - Decline to answer |
| What is your ethnicity? | - Non-Hispanic - Hispanic - Decline to answer |
| What is your marital status? | - Never married/never living like married - Married/living like married - Widowed - Divorced - Decline to answer |
| What is your household income? | - $0-14,000 - $14,001-$53,700 - $53,701-85,500 - $85,501-163,300 - $163,301-207,350 - $207,351-518,400 - $518,401 or more - Decline to answer |
| How many dependents (i.e., individuals who rely on your for major financial support) do you have? | - None - One - Two - Three - Four or more - Decline to answer |
| Are you the primary caretaker for anyone in your family or household | - No - Yes |
