# Supplementary material for: Healthcare providers’ perceived support from their organization is associated with lower burnout and anxiety amid the COVID-19 pandemic
Source: PLoS One. 2021 Nov 19;16(11):e0259858. doi: 10.1371/journal.pone.0259858 (PMC8604356; doi:10.1371/journal.pone.0259858)
Supplement: S1 Table — (DOCX) [file pone.0259858.s005.docx]

**S1 Table: Demographics of participants by randomization group (i.e., Survey version A vs. Survey version B)**

| **Demographic variable** | | **Survey A (No.)** | **Survey B (No.)** | **p-value** |
| --- | --- | --- | --- | --- |
| **Age^1^** | |  |  | .56 |
|  | <25 years old | 22 | 32 |  |
|  | 25-44 years old | 212 | 205 |  |
|  | 45-64 years old | 52 | 53 |  |
|  | >64 years old | 6 | 5 |  |
| Male | | 74 | 77 | .85 |
| Married or living like married | | 199 | 210 | .47 |
| Race^1,2^ | |  |  | .17 |
|  | White, European, or Middle Eastern | 246 | 242 |  |
|  | Black | 12 | 5 |  |
|  | Asian | 26 | 30 |  |
|  | Native Hawaiian or Other Pacific Islander | 0 | 1 |  |
|  | Other | 4 | 10 |  |
| Non-Hispanic ethnicity^2^ | | 274 | 277 | .96 |
| Income^1,2^ | |  |  | .51 |
|  | $0-14,000 | 2 | 6 |  |
|  | $14,000-53,700 | 31 | 35 |  |
|  | $53,701-85,500 | 78 | 75 |  |
|  | $85,501-163,300 | 85 | 73 |  |
|  | $163,301-207,350 | 21 | 17 |  |
|  | $207,351-518,400 | 57 | 60 |  |
|  | $518,401 or more | 6 | 12 |  |
| Occupation |  |  |  | >.99 |
|  | Attending | 76 | 77 |  |
|  | Trainee | 95 | 96 |  |
|  | Advanced Practice Provider | 21 | 23 |  |
|  | Nursing staff | 55 | 54 |  |
|  | Other^3^ | 45 | 45 |  |
| Parental status^4^ | | 157 | 159 | >.99 |
| Primary caretaker^5^ | | 199 | 185 | .19 |

1 Categorization consistent with 2020 census.

2 Survey indicated categorical responses as optional, declining to answer was therefore not considered missing data.

3 Defined as respiratory therapist or patient care technician.

4 Defined as having one or more child for whom the participant is a guardian.

5 Defined as serving as a primary caretaker for another individual.

All categorical variables are compared with chi-squared testing.
