# Supplementary material for: Healthcare providers’ perceived support from their organization is associated with lower burnout and anxiety amid the COVID-19 pandemic
Source: PLoS One. 2021 Nov 19;16(11):e0259858. doi: 10.1371/journal.pone.0259858 (PMC8604356; doi:10.1371/journal.pone.0259858)
Supplement: S2 Table — (DOCX) [file pone.0259858.s006.docx]

**S2 Table: Demographics of participants included and excluded from primary analysis**

| **Demographic variable** | | **Primary Cohort** | **Excluded Cohort** | **p-value** |
| --- | --- | --- | --- | --- |
| Age^1^ | |  |  | .35 |
|  | <25 years old | 13 (8) | 13 (13) |  |
|  | 25-44 years old | 292 (73) | 69 (67) |  |
|  | 45-64 years old | 71 (18) | 20 (19) |  |
|  | >64 years old | 8 (2) | 1 (1) |  |
| Male | | 107 (27) | 61 (45) | .09 |
| Married or living like married | | 263 (65) | 60 (44) | .02 |
| Race^1,2^ | |  |  | .18 |
|  | White, European, or Middle Eastern | 335 (84) | 81 (79) |  |
|  | Black | 9 (2) | 7 (7) |  |
|  | Asian | 39 (10) | 10 (10) |  |
|  | Native Hawaiian or Other Pacific Islander | 1 (<1) | 0 |  |
|  | Other | 8 (2) | 6 (4) |  |
|  | Declined to answer | 8 (2) | 1 (1) |  |
| Non-Hispanic ethnicity^2^ | | 9 (2) | 6 (4) | .23 |
| Income^1,2^ | |  |  | .01 |
|  | $0-14,000 | 5 (1) | 2 (2) |  |
|  | $14,000-53,700 | 34 (8) | 19 (19) |  |
|  | $53,701-85,500 | 102 (25) | 29 (21) |  |
|  | $85,501-163,300 | 110 (27) | 25 (18) |  |
|  | $163,301-207,350 | 26 (5) | 7 (7) |  |
|  | $207,351-518,400 | 94 (23) | 9 (9) |  |
|  | $518,401 or more | 13 (3) | 4 (4) |  |
|  | Declined to answer | 18 (4) | 7 (7) |  |
| Occupation |  |  |  | .098 |
|  | Attending | 115 (29) | 21 (15) |  |
|  | Trainee | 139 (35) | 34 (25) |  |
|  | Advanced Practice Provider | 31 (8) | 5 (4) |  |
|  | Nursing staff | 62 (15) | 20 (15) |  |
|  | Other^3^ | 55 (14) | 23 (17) |  |
| Parental status^4^ | | 225 (56) | 58 (43) | .14 |
| Primary caretaker^5^ | | 125 (31) | 33 (24) | .29 |

1 Categorization consistent with 2020 census.

2 Survey indicated categorical responses as optional, declining to answer was therefore not considered missing data.

3 Defined as respiratory therapist or patient care technician.

4 Defined as having one or more child for whom the participant is a guardian.

5 Defined as serving as a primary caretaker for another individual.

All categorical variables are compared with chi-squared testing.
