# Supplementary material for: Healthcare providers’ perceived support from their organization is associated with lower burnout and anxiety amid the COVID-19 pandemic
Source: PLoS One. 2021 Nov 19;16(11):e0259858. doi: 10.1371/journal.pone.0259858 (PMC8604356; doi:10.1371/journal.pone.0259858)
Supplement: S3 Table — (DOCX) [file pone.0259858.s007.docx]

**S3 Table: Subgroup analyses investigating whether association between perceived organizational support and outcomes of interest (i.e., burnout, anxiety) differ over categories of covariates**

|  |  | **Risk of burnout^1^** | | | **Anxiety^1^** | | |
| --- | --- | --- | --- | --- | --- | --- | --- |
| **Variable** | **Subgroup** | **Coeff.** | **95% CI** | **p-value of interaction** | **Coeff.** | **95% CI** | **p-value of interaction** |
| **COVID-19 symptoms** ^1^ (Ref., No) |  |  |  | .38 |  |  | .13 |
|  | Yes, $\Delta$ from No | 0.03 | -0.04, 0.10 |  | -0.03 | -0.07, 0.01 |  |
| **Time off for illness**^1^ (Ref., No) |  |  |  | .29 |  |  | .54 |
|  | Yes, $\Delta$ from No | -0.05 | -0.04, 0.15 |  | -0.04 | -0.08, 0.04 |  |
| **Strain on relationships**^1^ (Ref., No) |  | | | .26 |  |  | .027 |
|  | Yes, $\Delta$ from No | 0.03 | -0.02, 0.08 |  | -0.04 | -0.07, 0.00 |  |
| **Occupation** (Ref., Other^3^) |  | | | .87 |  |  | .001 |
|  | Attending, $\Delta$ from Other^3^ | 0.03 | -0.05, 0.12 |  | 0.06 | 0.01, 0.11 |  |
|  | Trainee, $\Delta$ from Other^3^ | 0.04 | -0.04, 0.12 |  | 0.06 | 0.01, 0.11 |  |
|  | Advanced practice provider, $\Delta$ from Other^3^ | 0.04 | -0.08, 0.17 |  | 0.11 | 0.04, 0.19 | .001 |
|  | Nursing staff, $\Delta$ from Other^3^ | 0.01 | -0.08, 0.10 |  | 0.00 | -0.06, 0.05 |  |
| **Hospital type**^6^ (Ref., Community) |  | | | .94 |  |  | .52 |
|  | Academic, $\Delta$ from Community | 0.00 | -0.06, 0.05 |  | -0.01 | -0.02, 0,04 |  |
| **Age^2^** (Ref., 45+) |  | | | .45 |  | | .71 |
|  | <24, $\Delta$ from 45+ | -0.01 | -0.09, 0.12 |  | -0.00 | -0.06, 0.07 |  |
|  | 25-44, $\Delta$ from 45+ | -0.03 | -0.09, 0.03 |  | -0.01 | 0.05, 0.07 |  |
| **Sex** (Ref., Female) |  | | | .17 |  | | .059 |
|  | Male, $\Delta$ from Female | 0.04 | -0.02, 0.10 |  | -0.03 | 0.00, 0.07 |  |
| **Married/living like married** (Ref., No) |  | | | .76 |  | | .64 |
|  | Yes, $\Delta$ from No | 0.01 | -0.05, 0.06 |  | -0.01 | -0.03, 0.04 |  |
| **White race^2^** (Ref., non-white) |  | | | .093 |  | | .083 |
|  | White, $\Delta$ from non-white | -0.06 | -0.13, 0.01 |  | -0.04 | -0.08, -0.01 |  |
| **Hispanic** (Ref., non-Hispanic) |  | | | .93 |  | | .059 |
|  | Hispanic, $\Delta$ from non-Hispanic | 0.01 | -0.27, 0.30 |  | 0.16 | -0.01, 0.33 |  |
| **Income^2^** (Ref., $163,301+) |  | | | .501 |  | | .016 |
|  | $0-53,000, $\Delta$ from $163,301+ | -0.05 | -0.14, 0.05 |  | -0.06 | -0.11, 0.00 | .64 |
|  | $53,001-85,500, $\Delta$ from $163,301+ | -0.05 | -0.12, 0.02 |  | -0.06 | -0.11, -0.02 |  |
|  | $85,501-163,300, $\Delta$ from $163,301+ | -0.02 | -0.09, 0.05 |  | -0.02 | -0.07, 0.02 |  |
| **Parental status**^4^ (Ref., No) |  | | | .91 |  | | .61 |
|  | Yes, $\Delta$ from No | 0.00 | -0.06, 0.05 |  | 0.01 | -0.02, 0.04 |  |
| **Primary caretaker**^5^ (Ref., No) |  | | | .78 |  | | .96 |
|  | Yes, $\Delta$ from No | -0.01 | -0.07, 0.05 |  | 0.00 | -0.04, 0.03 |  |

1 Repeated measures evaluated at each survey timepoint; Proportion of COVID-19 cases per maximal bed capacity at the identified hospital of employment by the participants

2 Categorization consistent with 2020 census. Categories consolidated if reported by <5% of respondents.

3 Defined as respiratory therapist or patient care technician

4 Defined as having one or more child for whom the participant is a guardian

5 Defined as serving as a primary caretaker for another individual

6 Defined as a tertiary care hospital that is organizationally integrates with a medical school and/or residency program

Displayed are the coefficients of the interactions terms, to be interpreted as the difference in the associaton between perceived organizational support and outcomes of interest, when compared to the category reference.
