# Supplementary material for: Healthcare providers’ perceived support from their organization is associated with lower burnout and anxiety amid the COVID-19 pandemic
Source: PLoS One. 2021 Nov 19;16(11):e0259858. doi: 10.1371/journal.pone.0259858 (PMC8604356; doi:10.1371/journal.pone.0259858)
Supplement: S4 Table — (DOCX) [file pone.0259858.s008.docx]

**S4 Table: Univariate associations between variables and outcomes of interest, sensitivity analysis using all time points**

|  | | **Risk of burnout** | | | **Anxiety** | | |
| --- | --- | --- | --- | --- | --- | --- | --- |
|  |  | **Coeff.** | **95% CI** | **P-Value** | **Coeff.** | **95% CI** | **P-Value** |
| Perceived organizational support | | -0.23 | -0.26, -0.21 | <.001 | -0.07 | -0.09 -0.06 | <.001 |
| Age, years (Reference, 45+) | |  |  |  |  |  |  |
|  | <25 | -1.23 | -2.69, 0.23 | .33 | -0.33 | -1.22, 0.55 | .46 |
|  | 25-44 | -0.41 | -1.25, 0.42 | .099 | 0.16 | -0.35, 0.66 | .54 |
| Male Sex | | -0.95 | -1.64, -0.26 | .007 | -1.04 | -1.46, -0.61 | <.001 |
| Occupation (Reference, other) | |  |  |  |  |  |  |
|  | Attending | -1.09 | -2.63, 0.46 | .17 | -1.82 | -2.77, -0.87 | <.001 |
|  | Trainee (resident/fellow) | -0.56 | -1.88, 0.76 | .41 | -1.22 | -2.02, -0.42 | .003 |
|  | Advanced practice provider | -1.13 | -2.68, 0.43 | .16 | -0.76 | -1.70, 0.18 | .12 |
|  | Nursing staff | -0.99 | -2.21, 0.24 | .11 | -0.51 | -1.25, 0.24 | .18 |
| Income (Reference, $163,301+) | |  |  |  |  |  |  |
|  | $0-53,000 | 0.23 | -1.29, 1.75 | .77 | 0.25 | -0.68, 1.18 | .60 |
|  | $53,701-85,500 | 0.26 | -0.86, 1.39 | .65 | 0.20 | -0.49, 0.88 | .57 |
|  | $85,501-163,300 | -0.02 | -1.09, 1.05 | .97 | -0.30 | -0.95, 0.35 | .36 |
| Parent status | | -1.08 | -1.78, -0.39 | .002 | -0.58 | -1.01, -0.16 | .01 |
| COVID-19 symptoms | | 2.44 | 1.52, 3.37 | <.001 | 0.97 | 0.41, 1.53 | .001 |
| Time taken off for illness | | 0.40 | -0.96, 1.76 | .57 | 0.49 | -0.34, 1.31 | .25 |
| Relationship strain | | 3.80 | 3.21, 4.39 | <.001 | 1.87 | 1.51, 2.23 | <.001 |
| Survey timeframe (Reference, April) | |  |  |  |  |  |  |
|  | May | 0.18 | -0.49, 0.86 | .59 | -0.43 | -0.89, 0.06 | .06 |
|  | June | -0.27 | -0.96, 0.42 | .45 | -0.41 | -0.83, 0.02 | .06 |
| COVID-19 Caseload | | -0.03 | -0.21, 0.15 | .55 | 0.03 | -0.09, 0.15 | .62 |
| Intercept | | 31.82 | 29.91, 33.73 | <.001 | 11.68 | 10.40, 12.96 | <.001 |

1 Categorization consistent with 2020 census. Categories consolidated if reported by <5% of respondents.

2 Defined as respiratory therapist or patient care technician

3 Defined as a tertiary care hospital that is organizationally integrates with a medical school and/or residency program

4 Defined as having one or more child for whom the participant is a guardian

5 Defined as serving as a primary caretaker for another individual

Analysis was performed for all six survey time points using a linear mixed model with fixed effects accounting for time, perceived organizational support, variables that were found to be associated with the outcome in the univariate analysis, and random hospital and participant effect
