# Supplementary material for: Healthcare providers’ perceived support from their organization is associated with lower burnout and anxiety amid the COVID-19 pandemic
Source: PLoS One. 2021 Nov 19;16(11):e0259858. doi: 10.1371/journal.pone.0259858 (PMC8604356; doi:10.1371/journal.pone.0259858)
Supplement: S5 Table — (DOCX) [file pone.0259858.s009.docx]

**S5 Table: Mediation Analysis, 1^st^ Survey (April 2020)**

**
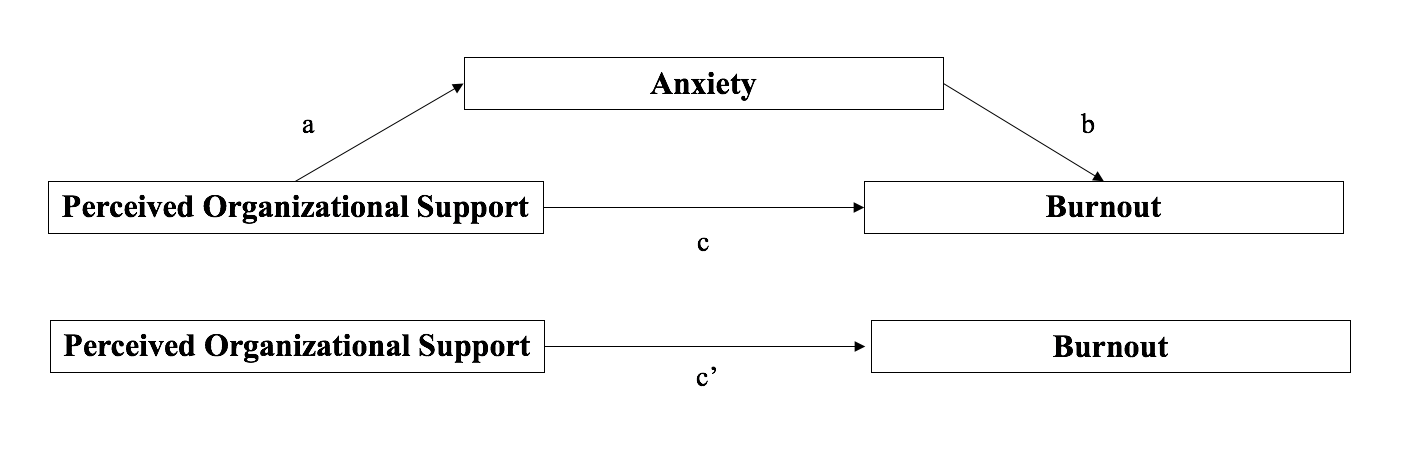
**

| **Variable** | | **Pathway a** | **Pathway c** | **Pathway c’** |
| --- | --- | --- | --- | --- |
|  | | Coeff. (95% CI); p-value | Coeff. (95% CI); p-value | Coeff.; 95% CI; p-value |
| Perceived organizational support | | -0.07 (-0.10, -0.04); <.001 | -0.17 (-0.21, -0.13); <.001 | -0.23 (-0.28, -0.19); <.001 |
| Anxiety | | -- | 0.86 (0.71, 1.00); <.001 | -- |
| Age | |  |  |  |
|  | ≤24 | 0.14 (-1.72, 1.44); .86 | 2.29 (-4.47, -0.12); .039 | 2.41 (-4.98, 0.15); .065 |
|  | 25-44 | 0.06 (-1.39, 1.50); .94 | 1.56 (-0.43, 3.55); .12 | 1.61 (-0.74, 3.95); .18 |
|  | 45+ | -- | -- | -- |
| Male | | -1.36 (-2.11, -0.61); <.001 | -0.25 (-1.30, 0.80); .64 | -1.42 (-2.63, -0.21); .022 |
| Married/living like married | | 1.09 (0.19, 2.00); .18 | -1.05 (-2.30, 0.20); .10 | -0.11 (-1.57, 1.36); .88 |
| Income | | -- | -- | -- |
|  | +$0-53,000 | -0.77 (-0.95, 2.49); .38 | 0.80 (-3.17, 1.57); .51 | 0.14 (2.93, 2.65); .92 |
|  | $53,701-85,500 | -0.83 (-2.29, 0.62); .26 | 1.03 (-0.98, 3.04); .31 | 0.32 (-2.05, 2.68); .79 |
|  | $85,501-163,300 | 0.56 (-0.80,1.92);.42 | -0.22 (-2.09, 1.66); .82 | 0.26 (-1.94, 2.47); .81 |
|  | $163,301 | -- | -- | -- |
| Occupation | |  |  |  |
|  | Attending | 1.89 (-3.53, -0.26); .024 | -0.21 (-2.47, 2.06); .86 | -1.83 (-4.48, 0.82); .18 |
|  | Resident/fellow | 0.84 (-0.57, 2.26); .24 | -0.93 (-2.88, 1.02); .35 | -0.20 (-2.49, 2.09); .86 |
|  | Advanced practice provider | 1.42 (-0.19, 3.02); .083 | -2.21 (-4.43, 0.01); .051 | -0.99 (-3.59, 1.61); .45 |
|  | Nurse | 0.46 (-0.75, 1.66); .46 | 0.58 (-1.09, 2.24); .50 | 0.97 (-0.99, 2.92); .33 |
|  | Other | -- | -- | -- |
| No Parental status | | 0.58 (-0.18, 1.35); .13 | 0.32 (-0.74, 1.37); .55 | 0.82 (-0.42, 2.05); .19 |
| COVID-19 symptoms | | 0.80 (-0.16, 1.75); .10 | 2.23 (0.93, 3.57); <.001 | 2.94 (1.39, 4.49); <.001 |
| Time taken off for illness | | 0.61 (-0.73, 1.94); .37 | 0.47 (-1.37, 2.31); .62 | 0.99 (-1.17, 3.15); .37 |
| Relationship strain | | 2.23 (1.61, 2.85); <.001 | 1.88 (0.97, 2.79); <.001 | 3.80 (2.79, 4.81); <.001 |
