# Supplementary material for: Healthcare providers’ perceived support from their organization is associated with lower burnout and anxiety amid the COVID-19 pandemic
Source: PLoS One. 2021 Nov 19;16(11):e0259858. doi: 10.1371/journal.pone.0259858 (PMC8604356; doi:10.1371/journal.pone.0259858)
Supplement: S6 Table — (DOCX) [file pone.0259858.s010.docx]

**S6 Table: Mediation Analysis, 2^nd^ Survey (May 2020)**

**
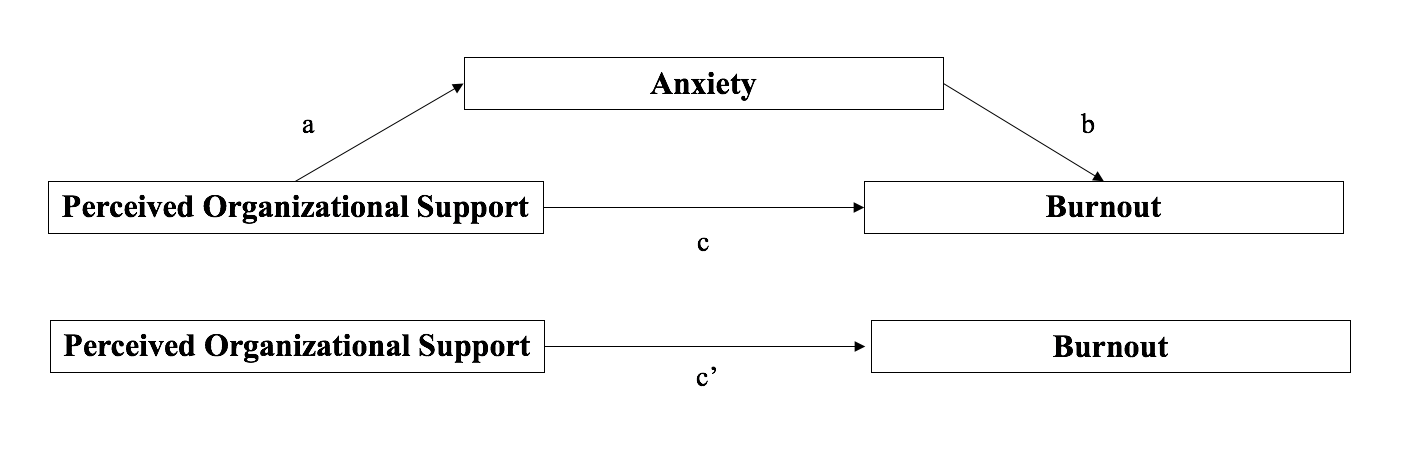
**

| **Variable** | | **Pathway a** | **Pathway c** | **Pathway c’** |
| --- | --- | --- | --- | --- |
|  | | Coeff. (95% CI); p-value | Coeff. (95% CI); p-value | Coeff.; 95% CI; p-value |
| Perceived organizational support | | -0.06 (-0.09, -0.04); <.001 | -0.16 (-0.20, -0.12); <.001 | -0.21 (-0.26, -0.17); <.001 |
| Anxiety | | -- | 0.77 (0.62, 0.91); <.001 | -- |
| Age | |  |  |  |
|  | ≤24 | -1.26 (-0.38, 2.91); .13 | 1.39 (-3.71, 0.93 ); .24 | 0.42 (-3.05, 2.21); .75 |
|  | 25-44 | -0.52 (-2.03, 0.99); .50 | 0.97 (1.16, 3.09); 0.37 | 0.42 (-2.21, 3.06); 0.75 |
|  | 45+ | -- | -- | -- |
| Male | | -0.92 (-1.69, -0.15); .019 | 0.48 (-0.61, 1.57); .39 | -0.23 (-1.46, 1.00); .72 |
| Married/living like married | | 0.83 (-0.10, 1.75); .079 | -0.83 (-2.13, 0.47); .21 | -0.19 (-1.67, 1.28); .80 |
| Income | | -- | -- | -- |
|  | $0-53,000 | 0.13 (-1.29, 1.56); .85 | 0.26 (-2.79, 2.27); .84 | -0.36 (-2.52, 3.24); .80 |
|  | $53,701-85,500 | -0.65 (-2.18, 0.88); .45 | 0.86 (-1.30, 3.01); .43 | 0.36 (-2.09. 2.81); .77 |
|  | $85,501-163,300 | -0.82 (-2.62, 0.99); .37 | -0.34 (-2.34, 1.66); .74 | -0.24 (-2.52, 2.05); .83 |
|  | $163, 301+ | -- | -- | -- |
| Occupation | |  |  |  |
|  | Attending | 0.10 (-1.16, 1.35); .88 | 0.56 (-1.84, 2.96); .65 | 0.04 (-2.69, 2.77); .98 |
|  | Resident/fellow | 0.86 (-1.16, 1.35); .88 | -1.43 (-3.51, 0.66); .18 | -0.96 (-3.33, 1.41); .43 |
|  | Advanced practice provider | 0.61 (-0.87, 2.10); .42 | -0.34 (-2.65, 1.97); .77 | 0.32 (-2.31, 2.94); .81 |
|  | Nurse | 0.67 (-0.87, 2.10); .44 | -0.24 (-2.01, 1.52); .79 | -0.17 (-2.18, 1.83); .87 |
|  | Other | -- | -- | -- |
| No Parental status | | 0.84 (0.04, 1.65); .039 | 1.04 (-0.09, 2.18); .07 | 1.69 (0.41, 2.97); .010 |
| COVID-19 symptoms | | 1.13 (0.08. 2.18); .035 | 2.94 (1.46, 4.42)l <.001 | 3.81 (2.13, 5.48); <.001 |
| Time taken off for illness | | 0.79 (-0.79, 2.37); .33 | -0.65 (-2.88, 1.57); .56 | -0.05 (-2.58, 2.48); .97 |
| Relationship strain | | 1.61 (0.94, 2.29); <.001 | 2.51 (1.53, 3.48); <.001 | 3.74 (2.67, 4.82); <.001 |
