# Supplementary material for: Healthcare providers’ perceived support from their organization is associated with lower burnout and anxiety amid the COVID-19 pandemic
Source: PLoS One. 2021 Nov 19;16(11):e0259858. doi: 10.1371/journal.pone.0259858 (PMC8604356; doi:10.1371/journal.pone.0259858)
Supplement: S7 Table — (DOCX) [file pone.0259858.s011.docx]

**S7 Table: Mediation Analysis, 3^rd^ Survey (June 2020)**

**
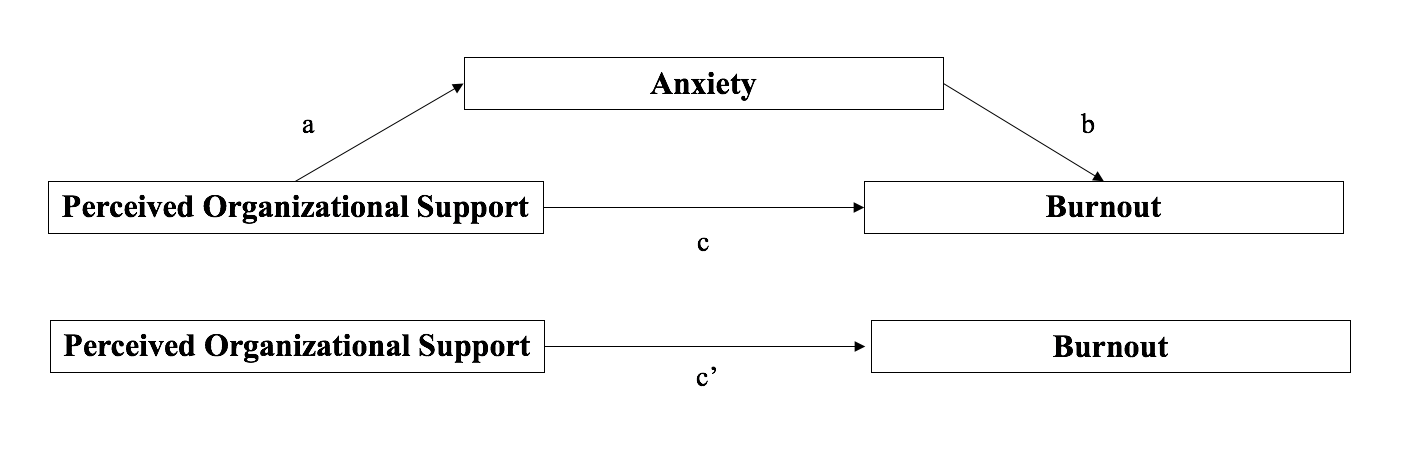
**

| **Variable** | | **Pathway a** | **Pathway c** | **Pathway c’** |
| --- | --- | --- | --- | --- |
|  | | Coeff. (95% CI); p-value | Coeff. (95% CI); p-value | Coeff.; 95% CI; p-value |
| Perceived organizational support | | -0.08 (-0.11, -0.06); <.001 | -0.18 (-0.22, -0.14); <.001 | -0.25 (-0.30, -0.20); <.001 |
| Anxiety | | -- | 0.84 (0.67, 1.01); <.001 | -- |
| Age | |  |  |  |
|  | ≤24 | 0.24 (-1.96, 1.48); .77 | 0.97 (-3.48, 1.54); .45 | 1.17 (-4.06, 1.73); .43 |
|  | 25-44 | -0.30 (-1.27, 1.86); .71 | 0.27 (-2.02, 2.55); .82 | 0.52 (-2.12, 3.15); .70 |
|  | 45+ | -- | -- | -- |
| Male | | -1.19 (1.99, -0.40); .003 | 0.51 (-0.66, 1.68); .39 | -0.49 (-1.83, 0.84); .47 |
| Married/living like married | | 1.06 (0.10, 2.03); .031 | -0.34 (-1.76, 1.07); .64 | 0.55 (-1.07, 2.17); .50 |
| Income | | -- | -- | -- |
|  | $0-53,000 | 0.43 (-2.22, 1.36); .64 | 0.22 (-2.82, 2.39); .87 | 0.58 (-3.59. 2.43); .71 |
|  | $53,701-85,500 | -0.77 (-2.31, 0.78); .33 | 0.44 (-1.79, 2.73); .68 | -0.17 (-2.77, 2.43); .90 |
|  | $85,501-163,300 | -0.11 (-1.56, 1.34); .88 | 0.59 (-1.52, 2.71); .581 | 0.50 (1.94, 2.94); .69 |
|  | $163,301+ | -- | -- | -- |
| Occupation | |  |  |  |
|  | Attending | 2.57 (-4.32, -0.82); .004 | 0.50 (-3.09, 2.08); .70 | 2.67 (-5.61, 0.28); .076 |
|  | Resident/fellow | 2.09 (0.61, 3.56); .010 | -0.26 (-2.44, 1.92); .82 | 1.50 (-0.98, 3.98); .24 |
|  | Advanced practice provider | 1.09 (-0.52, 2.71); .18 | -0.15 (2.51, 2.21); .90 | 0.77 (-1.95, 3.49); .58 |
|  | Nurse | 1.09 (-0.19, 2.37); .01 | -0.41 (-2.28, 1.47); .67 | 0.51 (-1.64, 2.66); .64 |
|  | Other | -- | -- | -- |
| No Parental stauts | | 0.66 (-0.15, 1.47); .11 | 0.67 (-0.51, 1.86); .26 | 1.23 1-0.13, 2.59); .076 |
| COVID-19 symptoms | | 0.80 (-0.32, 1.92); .16 | 0.28 (-1.36, 1.91); .74 | 0.95 (-0.93, 2.83); .31 |
| Time taken off for illness | | 0.20 (-1.51, 1.92); .82 | -0.62 (-3.13, 1.88); .63 | -0.45 (-3.34, 2.43); .76 |
| Relationship strain | | 2.08 (1.40, 2.77); <.001 | 2.40 (1.33 3.46); <.001 | 4.15 (2.99, 5.31); <.001 |
