# Supplementary material for: Healthcare providers’ perceived support from their organization is associated with lower burnout and anxiety amid the COVID-19 pandemic
Source: PLoS One. 2021 Nov 19;16(11):e0259858. doi: 10.1371/journal.pone.0259858 (PMC8604356; doi:10.1371/journal.pone.0259858)
Supplement: S1 Fig — (DOCX) [file pone.0259858.s012.docx]

**S1 Fig**. **Repeated measure data and COVID-19 caseload over time**

Line graphs represent repeated measures which may associate with COVID-19 (Panel A), the psychosocial constructs (Panel B), and COVID-19 Caseload (Panel C) throughout the course of the study. In Panel A, the proportion of respondents included (dark gray) and excluded (light gray) from the primary analysis were calculated overtime. In Panel B, the Y-axis range the minimal to maximal cumulative score for each scale, the symbols indicate mean score for those included and excluded from the primary cohort and analysis with associated standard errors. In Panel C, the symbol is as the percentage of patients with known COVID-19 per possible number of system wide hospital beds with associated 95% confidence intervals.
