# Supplementary material for: Healthcare providers’ perceived support from their organization is associated with lower burnout and anxiety amid the COVID-19 pandemic
Source: PLoS One. 2021 Nov 19;16(11):e0259858. doi: 10.1371/journal.pone.0259858 (PMC8604356; doi:10.1371/journal.pone.0259858)
Supplement: Attachment — Submitted filename: PONE-D-21-19123.pdf [file pone.0259858.s014.pdf]

PLOS ONE

Healthcare providers’ perceived support from their organization is associated with lower burnout amid the COVID-19 pandemic

--Manuscript Draft--

**Manuscript Number:**	PONE-D-21-19123
**Article Type:**	Research Article
**Full Title:**	Healthcare providers’ perceived support from their organization is associated with lower burnout amid the COVID-19 pandemic
**Short Title:**	Provider burnout during COVID-19
**Corresponding Author:**	Sara Myers<br><br>UNITED STATES
**Keywords:**	Burnout; COVID-19; Perceived Organizational Support; Anxiety
**Abstract:**	Background<br><br>Professional burnout represents a significant threat to the American healthcare system. Organizational and individual factors may increase healthcare providers’ susceptibility or resistance to burnout. We hypothesized that during the COVID-19 pandemic, 1) higher levels of perceived organizational support (POS) are associated with lower risk for burnout and anxiety, and 2) anxiety mediates the association between POS and burnout.<br><br>Methods<br><br>In this longitudinal prospective study, we surveyed healthcare providers employed full-time at a large, multihospital healthcare system monthly over 6 months (April to November 2020). Participants were randomized using a 1:1 allocation stratified by provider type, gender, and academic hospital status to receive one of two versions of the survey instrument formulated with different ordering of the measures to minimize response bias due to context effects. The exposure of interest was POS measured using the validated 8-item Survey of POS (SPOS) scale. Primary outcomes of interest were anxiety and risk for burnout as measured by the validated 10-item Burnout scale from the Professional Quality (Pro-QOL) instrument and 4-item Emotional Distress-Anxiety short form of the Patient Reported Outcome Measurement Information System (PROMIS) scale, respectively. Linear mixed models evaluated the associations between POS and both burnout and anxiety. A mediation analysis evaluated whether anxiety mediated the POS-burnout association.<br><br>Results<br><br>Of the 538 participants recruited, 402 (75%) were included in the primary analysis. 55% of participants were physicians, 73% 25-44 years of age, 73% female, 83% White, and 44% had [[EQUATION]] 1 dependent. Higher POS was significantly associated with a lower risk for burnout (-0.23; 95% CI -0.26, -0.21; p<0.001) and lower degree of anxiety (-0.07; 95% CI -0.09, -0.06; p=0.010). Anxiety mediated the associated between POS and burnout (direct effect -0.17; 95% CI -0.21, -0.13; p<0.001; total effect -0.23; 95% CI -0.28, -0.19; p<0.001).<br><br>Conclusion<br><br>During a health crisis, increasing the organizational support perceived by healthcare employees may reduce the risk for burnout through a reduction in anxiety. Improving the relationship between healthcare organizations and the individuals they employ may reduce detrimental effects of psychological distress among healthcare providers and ultimately improve patient care.
**Order of Authors:**	Sara Myers
	Katherine Moll Reitz

Powered by Editorial Manager® and ProduXion Manager® from Aries Systems Corporation

	Lauren Terhorst
	Clair Smith
	Insiyah Campwala
	Stephanie Downs-Canner
	Emilia Diego
	Galen Switzer
	Matthew Rosengart
	Maryanna Owoc
**Opposed Reviewers:**	
**Additional Information:**	
**Question**	**Response**
**Financial Disclosure**<br><br>Enter a financial disclosure statement that describes the sources of funding for the work included in this submission. Review the submission guidelines for detailed requirements. View published research articles from *PLOS ONE* for specific examples.<br><br>This statement is required for submission and **will appear in the published article** if the submission is accepted. Please make sure it is accurate.<br><br>**Unfunded studies**<br>Enter: *The author(s) received no specific funding for this work.*<br><br>**Funded studies**<br>Enter a statement with the following details:<br>• Initials of the authors who received each award<br>• Grant numbers awarded to each author<br>• The full name of each funder<br>• URL of each funder website<br>• Did the sponsors or funders play any role in the study design, data collection and analysis, decision to publish, or preparation of the manuscript?<br>• **NO** - Include this sentence at the end of your statement: *The funders had no role in study design, data collection and analysis, decision to publish, or preparation of the manuscript.*<br>• **YES** - Specify the role(s) played.<br><br>* typeset	Dr. Katherine Reitz was supported in part by the National Heart, Lung, and Blood Institute (5T32HL0098036) and National Institutes of Health (L30AG064730).

**Competing Interests**<br><br>Use the instructions below to enter a competing interest statement for this submission. On behalf of all authors, disclose any competing interests that could be perceived to bias this work—acknowledging all financial support and any other relevant financial or non-financial competing interests.<br><br>This statement is required for submission and **will appear in the published article** if the submission is accepted. Please make sure it is accurate and that any funding sources listed in your Funding Information later in the submission form are also declared in your Financial Disclosure statement.<br><br>View published research articles from *PLOS ONE* for specific examples.<br><br>**NO authors have competing interests**<br><br>Enter: *The authors have declared that no competing interests exist.*<br><br>**Authors with competing interests**<br><br>Enter competing interest details beginning with this statement:<br><br>*I have read the journal's policy and the authors of this manuscript have the following competing interests: [insert competing interests here]*<br><br>* typeset	No author have competing interests.
**Ethics Statement**<br><br>Enter an ethics statement for this submission. This statement is required if the study involved:<br><br>• Human participants<br>• Human specimens or tissue<br>• Vertebrate animals or cephalopods	This study involved human participants. This study was approved by the University of Pittsburgh Institutional Review Board (STUDY20040051). Written consent was obtained.

• Vertebrate embryos or tissues<br>• Field research<br><br>Write "N/A" if the submission does not require an ethics statement.<br><br>General guidance is provided below. Consult the submission guidelines for detailed instructions. **Make sure that all information entered here is included in the Methods section of the manuscript.**	

Powered by Editorial Manager® and ProduXion Manager® from Aries Systems Corporation

**Format for specific study types**<br><br>**Human Subject Research (involving human participants and/or tissue)**<br>• Give the name of the institutional review board or ethics committee that approved the study<br>• Include the approval number and/or a statement indicating approval of this research<br>• Indicate the form of consent obtained (written/oral) or the reason that consent was not obtained (e.g. the data were analyzed anonymously)<br><br>**Animal Research (involving vertebrate animals, embryos or tissues)**<br>• Provide the name of the Institutional Animal Care and Use Committee (IACUC) or other relevant ethics board that reviewed the study protocol, and indicate whether they approved this research or granted a formal waiver of ethical approval<br>• Include an approval number if one was obtained<br>• If the study involved *non-human primates*, add *additional details* about animal welfare and steps taken to ameliorate suffering<br>• If anesthesia, euthanasia, or any kind of animal sacrifice is part of the study, include briefly which substances and/or methods were applied<br><br>**Field Research**<br><br>Include the following details if this study involves the collection of plant, animal, or other materials from a natural setting:<br>• Field permit number<br>• Name of the institution or relevant body that granted permission	
**Data Availability**<br><br>Authors are required to make all data underlying the findings described fully available, without restriction, and from the time of publication. PLOS allows rare exceptions to address legal and ethical concerns. See the PLOS Data Policy and FAQ for detailed information.	No - some restrictions will apply

A Data Availability Statement describing where the data can be found is required at submission. Your answers to this question constitute the Data Availability Statement and **will be published in the article**, if accepted.<br><br>**Important:** Stating 'data available on request from the author' is not sufficient. If your data are only available upon request, select 'No' for the first question and explain your exceptional situation in the text box.<br><br>Do the authors confirm that all data underlying the findings described in their manuscript are fully available without restriction?	
**Describe where the data may be found in full sentences. If you are copying our sample text, replace any instances of XXX with the appropriate details.**<br><br>• If the data are **held or will be held in a public repository**, include URLs, accession numbers or DOIs. If this information will only be available after acceptance, indicate this by ticking the box below. For example: *All XXX files are available from the XXX database (accession number(s) XXX, XXX.*).<br>• If the data are all contained **within the manuscript and/or Supporting Information files**, enter the following: *All relevant data are within the manuscript and its Supporting Information files.*<br>• If neither of these applies but you are able to provide **details of access elsewhere**, with or without limitations, please do so. For example:<br><br>*Data cannot be shared publicly because of [XXX]. Data are available from the XXX Institutional Data Access / Ethics Committee (contact via XXX) for researchers who meet the criteria for access to confidential data.*<br><br>*The data underlying the results presented in the study are available from (include the name of the third party*	Data will be made available by written request to principle investigator.

and contact information or URL).<br>• This text is appropriate if the data are owned by a third party and authors do not have permission to share the data.<br><br>* typeset	
Additional data availability information:	Tick here if the URLs/accession numbers/DOIs will be available only after acceptance of the manuscript for publication so that we can ensure their inclusion before publication.

University of Pittsburgh
Department of Surgery
School of Medicine

June 10, 2021

Emily Chenette, PhD
PLOS ONE
Re: Healthcare providers' perceived support from their organization is associated with lower burnout amid the COVID-19 pandemic

Dear Dr. Chenette:

We are pleased to submit our manuscript, referenced above, for review by the *PLOS ONE.*

As you know, healthcare providers have been disproportionately affected by COVID-19, which has translated to detrimental consequences for mental health, and, specifically, increased burnout. In addition to the consequences to providers and their patients, burnout represents a serious threat to the US economy. In this longitudinal prospective survey study of healthcare providers across a large, multi-hospital healthcare system, we show, first, that the degree to which an individual feels valued and supported by their employer, i.e., perceived organizational support (POS), influences burnout under the circumstances of the pandemic, and second, that anxiety mediates this relationship. Our hope it that this work, which employs rigorous survey-based methodology, will be the impetus for further investigation and implementation of interventions aimed at reducing provider burnout. Ultimately, preserving the well-being of healthcare providers is paramount for providing optimal patient care. In particular, facilitating a strong relationship between healthcare organizations and their employees is critical to delivering effective healthcare in times of crisis.

This manuscript has not been previously published and is not under consideration elsewhere. All authors have no conflicts of interest to report, including specific financial interests, relationships, or affiliations relevant to this manuscript.

Thank you in advance for your consideration. We sincerely appreciate this opportunity.

Very best regards,

Sara P. Myers, MD, PhD

Manuscript file

Click here to access/download;Manuscript;Burnout_Manuscript_6_10_PLoS.d

Healthcare providers' perceived support from their organization is associated with lower burnout amid the COVID-19 pandemic

Short title: Provider burnout during COVID-19

Katherine M. Reitz MD MSc,[1] Lauren Terhorst PhD,[2,3] Clair N. Smith MS,[4] Insiyah Campwala BS,[5] Maryanna Owoc BS,[5] Stephanie M. Downs-Canner MD,[6] Emilia Diego MD,[1] Galen E. Switzer PhD,[3,7,8,9] Matthew R. Rosengart MD MPH,[1,3] Sara P. Myers MD PhD[1]

Author and Group Information

1 Department of Surgery, University of Pittsburgh, Pittsburgh, PA, USA
2 School of Health and Rehabilitation Sciences, University of Pittsburgh, PA, USA
3 Department of Clinical and Translational Science, University of Pittsburgh, PA, USA
4 Department of Orthopaedic Surgery, University of Pittsburgh, Pittsburgh, PA, USA
5 School of Medicine, University of Pittsburgh, Pittsburgh, PA, USA
6 Department of Surgery, University of North Carolina-Chapel Hill School of Medicine, Chapel Hill, NC
7 Department of General Medicine, University of Pittsburgh, Pittsburgh, PA, USA
8 Department of Psychiatry, University of Pittsburgh, Pittsburgh, PA
9 Center for Health Equity Research and Promotion, Veterans Affairs Pittsburgh Healthcare System, Pittsburgh, PA

Corresponding Author

Sara P. Myers, MD, PhD
Department of Surgery, UPMC
University of Pittsburgh School of Medicine
200 Lothrop St, F677 Presbyterian Hospital
Pittsburgh, PA 15213
Phone: (917) 602 9028
Email: myerssp@upmc.edu
ORCID: https://orcid.org/0000-0003-4318-3938

Keywords

Burnout, COVID-19, Perceived Organizational Support, Anxiety

Manuscript Word Count

2608/3000

Abstract

Background: Professional burnout represents a significant threat to the American healthcare system. Organizational and individual factors may increase healthcare providers' susceptibility or resistance to burnout. We hypothesized that during the COVID-19 pandemic, 1) higher levels of perceived organizational support (POS) are associated with lower risk for burnout and anxiety, and 2) anxiety mediates the association between POS and burnout.

Methods: In this longitudinal prospective study, we surveyed healthcare providers employed full-time at a large, multihospital healthcare system monthly over 6 months (April to November 2020). Participants were randomized using a 1:1 allocation stratified by provider type, gender, and academic hospital status to receive one of two versions of the survey instrument formulated with different ordering of the measures to minimize response bias due to context effects. The exposure of interest was POS measured using the validated 8-item Survey of POS (SPOS) scale. Primary outcomes of interest were anxiety and risk for burnout as measured by the validated 10-item Burnout scale from the Professional Quality (Pro-QOL) instrument and 4-item Emotional Distress-Anxiety short form of the Patient Reported Outcome Measurement Information System (PROMIS) scale, respectively. Linear mixed models evaluated the associations between POS and both burnout and anxiety. A mediation analysis evaluated whether anxiety mediated the POS-burnout association.

Results: Of the 538 participants recruited, 402 (75%) were included in the primary analysis. 55% of participants were physicians, 73% 25-44 years of age, 73% female, 83% White, and 44% had ≥1 dependent. Higher POS was significantly associated with a lower risk for burnout (-0.23; 95% CI -0.26, -0.21; p<0.001) and lower degree of anxiety (-0.07; 95% CI -0.09, -0.06; p=0.010). Anxiety mediated the associated between POS and burnout (direct effect -0.17; 95% CI -0.21, -0.13; p<0.001; total effect -0.23; 95% CI -0.28, -0.19; p<0.001).

Conclusion: During a health crisis, increasing the organizational support perceived by healthcare employees may reduce the risk for burnout through a reduction in anxiety. Improving the relationship between

healthcare organizations and the individuals they employ may reduce detrimental effects of psychological distress among healthcare providers and ultimately improve patient care.

Word count: 345/350

Introduction

Frontline healthcare providers have been disproportionately affected by coronavirus disease 2019 (COVID-19) in their places of employment.[1] The global health crisis incited by COVID-19 has had detrimental consequences for mental health and, specifically, may potentiate provider burnout.[1] Professional burnout, characterized by emotional exhaustion, career de-prioritization, and loss of self-efficacy, represents a significant threat to the American healthcare system.[1,2] While burnout has been described as a reaction to organizational factors such as work-related stress,[3] individual factors also increase susceptibility to burnout.[4] An individual's anxiety in response to crisis may be one such factor.[5] It is, however, unknown whether the degree to which an individual feels supported by his/her/their employer, i.e., perceived organizational support (POS), a key driver of job satisfaction and performance,[5] influences anxiety and burnout under circumstances of crisis. In this longitudinal prospective study of healthcare providers, we hypothesized that during the COVID-19 pandemic, 1) higher levels of POS are associated with lower burnout and anxiety, and 2) anxiety mediates the association between POS and burnout. Understanding the relationship between these constructs is paramount to designing interventions that preserve the psychological wellbeing of healthcare providers, a requisite for maintaining a healthy and productive workforce, and delivering optimal patient care.[6]

Methods

PARTICIPANTS, SETTING, STUDY DESIGN

A longitudinal prospective survey study of healthcare providers from 20 community and academic hospitals within a single healthcare system in Pennsylvania was conducted to determine the association of POS with burnout and anxiety. Via an online survey, validated measures were administered monthly for a six-month period (April 28 to October 11, 2020). This study was approved by the University of Pittsburgh Institutional Review Board (STUDY20040051).

Based on previous literature investigating burnout among healthcare providers during COVID-19, assuming 35% of providers experience burnout, a minimum of 88 respondents were required to evaluate the association between POS and burnout, allowing for a 0.1 acceptable margin of error and alpha of 0.05.[1] All full-time providers employed by the healthcare system were recruited for study participation via e-mail (Appendix A). E-mail addresses were obtained from an internal employee electronic directory (April 1-14, 2020). Attending physicians, physicians in training (i.e., residents, fellows), advanced practice providers (i.e., nurse practitioners, physician assistants), nurses, and other providers (i.e., respiratory therapists, patient care technicians) with sufficient exposure to work-place and institutional culture (i.e., >12 months of employment) were eligible for inclusion. As this study aims to assess reactions associated with increased risk of patient-care related COVID-19 exposure, healthcare workers whose primary patient contact was via telehealth were excluded. Participants were asked to verify aforementioned inclusion/exclusion criteria, consent to participation, and provide baseline demographic data via electronic enrollment form (Appendix B, C). Based on data obtained from this form, participants were then randomized using a 1:1 allocation stratified by provider type, gender, and academic hospital status to receive one of two versions of the survey instrument formulated with different ordering of the measures (Appendices D, E) to minimize response bias due to context effects.[7] Participants received the same version at all time points via an automated email providing a link to a

University of Pittsburgh RedCap database where de-identified data was collected, stored, and connected to the individuals' email for repeated measure. Each survey also assessed whether participants had experienced parameters that may be associated with COVID-19 and/or its detrimental emotional effects including yes/no questions regarding perceived illness, illness necessitating time off from work, and strain on personal relationships. As we speculated that providers' degree of stress might correlate with COVID-19-related hospital admissions, we determined monthly per hospital caseload for each participant. Caseload was defined as the proportion of COVID-19 admissions per total hospital beds, abstracted from the healthcare system.[8] Survey participation to completion was incentivized by the random selection of two $250 and 20 $25 VISA® and Starbucks® gift cards.

STUDY MEASURES

POS was assessed using the validated, unidimensional 8-item Survey of POS (SPOS) scale.[9,10] Items appraise the degree to which an individual agreed with statements describing support or commitment from their employer organization in the 30 days prior using a 7-point Likert scale (0=Strongly disagree, 6=Strongly agree). Risk for burnout was evaluated using the 10-item Burnout scale from the Professional Quality (Pro-QOL) scale. This instrument is a validated, multidimensional metric that assesses consequences of stress a provider may experience from exposure to patients in emotional or physical distress.[11,12] Participants reflect on the frequency with which they experience hopelessness or difficulty completing job-related tasks effectively in the prior 30-days using a 5-point Likert scale (1=Never, 5=Very often). General anxiety was assessed using the 4-item Emotional Distress-Anxiety short form of the validated, multidimensional Patient Reported Outcome Measurement Information System (PROMIS)[13] scale that evaluates the frequency to which anxiety-related symptoms were experienced in the prior 7-days using a 5-point Likert scale (1=Never, 5=Very often). Each individual scale quantifies the psychosocial construct by the summation of all responses to Likert items. For each scale,

higher total scores represent more of the psychosocial construct. Internal consistency of each scale or subscale are as follows: 8-item SPOS=0.93,[14] Burnout=0.84,[15] and Anxiety= 0.97.[16]

STATISTICAL ANALYSIS PLAN

Descriptive statistics were generated for demographics, summary scales, and additional variables of interest. As a means of managing missingness unlikely to be at random and due to loss to follow-up, we restricted our primary analysis to include survey time points with at least 75% participant retention.[17] Thus, participants who completed at least two of the three first surveys constituted the cohort included in the primary analysis. Demographic data and variables of interest were compared between participants receiving version A and B, and between cohorts included and excluded from the primary analysis.

The association of POS, burnout, and anxiety were examined using linear mixed models that included fixed effect of time and random effects for hospital and participant.[18] Multivariable models additionally adjusted for covariates that were significantly associated with burnout or anxiety on univariable analysis and COVID-19 caseload. Further exploratory analyses, agreed upon *a priori,* were performed to determine how demographic variables moderate associations between POS, anxiety, and burnout. Sensitivity analyses were performed to assess how associations in the primary analysis between POS and outcomes of interest may have differed when using data from all six survey administrations. We performed a mediation analysis for each of the first three survey administrations with 5000 bootstrapped samples to evaluate if anxiety was the mechanism through which POS transmits its effect on burnout.[19]

Analysis, completed with SAS 9.4 (SAS Institute Inc.; PROCESS macro) or PRISM 7.0 (GraphPad), and data presentation were compliant with the Strengthening the Reporting of Observational Studies in

Epidemiology guidelines (Appendix F).[20] Statistical significance was considered below a threshold p-value of 0.05.

Results

538 individuals completed the enrollment form. 516 were eligible for study inclusion and participated in survey 1. The primary analysis included data from 402 participants who completed surveys 1-3 (Figure 1). Demographic data are presented in Table 1. Demographics did not differ based on survey version (eTable 1). Compared to participants whose data was excluded from the primary analysis, a greater proportion of participants included were married or living like married, White persons, and had income ≥$163,301 (eTable 2). Those included in the primary analysis reported more perceived illness and time off from work, but less relationship strain than participants excluded (eFigure 1). Study measures (i.e., POS, risk of burnout, and anxiety) did not differ overtime or between groups. Age, gender, occupation, income, having dependents, perceiving illness, experiencing illness necessitating time off of work, and endorsing relationship strain were associated with either burnout or anxiety on univariate analysis and included in the adjusted model (Table 2).

Figure 1. Cohort Accrual.

Table 1. Enrollment questionnaire for participants included in primary analysis

Variable – No. (%)	Primary Cohort (N=402)
Age, years[1]	
<24	31 (8)
25-44	292 (73)
45-64	71 (18)
65+	8 (2)
Male	110 (27)
Married or living like married	263 (65)
Race[1,2]	
White American, European American, or Middle Eastern American	335 (83)
Black or African American	9 (2)
Asian American	39 (10)
Native Hawaiian or Other Pacific Islander	1 (0.3)
Other	8 (2)
Declined to answer	8 (2)
Hispanic ethnicity	9 (2)
Occupation	263 (65)
Attending physician	115 (29)
Trainee (resident/fellow)	139 (35)
Advanced practice provider	31 (8)
Nursing staff	62 (15)
Other staff[3]	55 (14)
Academic Hospital[4]	236 (59)
Income[1,2]	
$0-14,000	5 (2)
$14,001-53,000	34 (9)
$53,001-85,500	102 (25)
$85,501-163,300	110 (27)
$163,301-207,350	26 (7)
$207351-518,400	94 (23)
$518,041+	13 (3)
Declined to answer	18 (5)
Parent status[1,5]	
No dependents	225 (56)
1 dependent	66 (17)
2 dependents	57 (14)
3 dependents	35 (9)
4+ dependents	18 (5)
Primary caretaker status[6]	125 (31)

1 Categorization consistent with 2020 census.
2 Survey indicated categorical responses as optional, declining to answer was therefore not considered missing data.
3 Defined as respiratory therapist or patient care technician.
4 Defined as a tertiary care hospital that is organizationally integrates with a medical school and/or residency program.
5 Defined as having one or more child for whom the participant is a guardian.
6 Defined as serving as a primary caretaker for another individual.

Table 2. Univariate associations between variables and outcomes of interest in the primary cohort

	Burnout[1]			Anxiety[1]		
**Variable**	Coefficient	95% CI	p-value	Coefficient	95% CI	p-value
Age2, years			0.070			<0.001
≤24	1.87	(0.28, 3.45)		2.28	(1.41, 3.14)	
25-44	0.55	(-0.38, 1.48)		0.87	0.36, 1.38)	
45+	Ref			Ref		
Male sex	-1.49	(-2.33, -0.65)	0.001	-1.67	(-2.13, -1.22)	<0.001
Married/living like married	-0.50	(-1.27, 0.27)	0.200	-0.31	(-0.73, 0.12)	0.150
Race[2]						
White, European, Middle-Eastern	0.99	(-0.02, 2.00)	0.051	0.19	(-0.37, 0.75)	0.500
Hispanic Ethnicity	-0.77	(-3.26, 1.71)	0.540	0.77	(-0.58, 2.12)	0.260
Income[2]			0.001			<0.001
$0-53,000	1.93	(0.57, 3.28)		2.29	()1.55, 3.02)	
$53,701-85,500	1.82	(0.85, 2.79)		1.53	(1.00, 2.06)	
$85,501-163,300	0.90	(-0.06, 1.86)		0.72	(0.19, 1.24)	
$163,301+	Ref	Ref		Ref	Ref	
Occupation			<0.001			<0.001
Attending	-3.06	(-4.41, -1.72)		-3.08	(-3.75, -2.40)	
Resident/fellow	-1.85	(-3.57, -0.13)		-1.93	(-2.61, -1.25)	
Advanced practice provider	-1.76	(-3.44, -0.08)		-1.51	(-2.39, -0.64)	
Nursing staff	0.51	(-0.86, 1.88)		-0.09	(-0.82, 0.64)	
Other staff [3]	Ref	Ref		Ref	Ref	
Parent status[2,4]	-1.20	(-1.94. -0.46)	0.002	-0.97	(-1.37, 0.56)	<0.001
Primary caretaker[5]	-0.05	(-0.85, 0.75)	0.900	-0.01	(-0.45, 0.43+	0.960
Academic hospital[6]	-0.39	(-0.54, 0.60)	0.600	0.33	(-0.76, 1.42)	0.550
Perceived illness[1]	4.93	(4.00, 5.87)	<0.001	2.47	(1.96, 2.99)	<0.001
Time taken off for illness[1]	4.45	(2.95, 5.95)	<0.001	2.48	(1.66, 3.31)	<0.001
Relationship strain[1]	5.47	(4.81, 6.14)	<0.001	2.67	(2.29, 3.04)	<0.001
COVID-19 Caseload[1,7]	-0.01	(-0.20,0.19)	0.186	0.03	(-0.12, 0.18)	0.176

1 Repeated measures evaluated at each survey timepoint
2Categorization consistent with 2020 census. Categories consolidated if reported by <5% of respondents.
3 Defined as respiratory therapist or patient care technician
4 Defined as having one or more child for whom the participant is a guardian
5 Defined as serving as a primary caretaker for another individual
6 Defined as a tertiary care hospital that is organizationally integrates with a medical school and/or residency program
7 Proportion of COVID-19 cases per maximal bed capacity at the identified hospital of employment by the participants

Values are from linear mixed models with fixed effects accounting for time and the single variable of interest as well as random effects accounting for the hospital and participant.
Abbreviations: CI, confidence intervals.

BURNOUT

Higher POS was significantly associated with a lower risk for burnout (Table 3). Having dependents was associated with a lower risk of burn out. Perceiving illness, endorsing strain on personal relationships, and being nurse (as compared with respiratory therapists and patient care technicians as the reference category) were associated with a higher risk of burn out (Table 3). The association between higher POS and lower risk of burnout was consistently observed, both across all subgroups and in sensitivity analysis (eTable 3-4). In addition to the variables that were significantly associated with burnout in the primary analysis, male gender was significantly associated with reduced risk of burnout in the sensitivity analysis (eTable 4).

Table 3. Adjusted analysis between variables and outcomes of interest in the primary cohort

	Burnout[1]			Anxiety[1]		
	Coefficient	95% CI	p-value	Coefficient	95% CI	p-value
Perceived organizational [1]	-0.23	(-0.26, -0.21)	<.0001	-0.07	(-0.09, -0.06)	<0.001
Age2, years (Reference, 45+)						
<25	-0.42	(-1.29, 0.46)	0.350	0.20	(-0.33, 0.73)	0.460
25-44	-1.28	(-2.84, 0.28)	0.109	-0.58	(-1.02, -0.14)	0.009
Male Sex	-0.73	(-1.47, 0)	0.050	0.94	(0.50, 1.39)	<0.001
Occupation (Reference, other staff[3])						
Attending	-1.18	(-2.79, 0.44)	0.152	-2.08	(-3.06, -1.10)	<0.001
Trainee (resident/fellow)	-0.58	(-1.98, 0.82)	0.414	-1.44	(-2.28, -0.60)	0.001
Advanced practice provider	-1.25	(-2.87, 0.37)	0.129	-0.94	(-1.92, 0.05)	0.063
Nursing staff	-1.32	(-2.59, -0.04)	0.044	-0.74	(-1.51, 0.04)	0.063
Income (Reference, $163,301+)						
$0-53,000	-0.07	(-1.67, 1.53)	0.931	-0.05	(-1.03, 0.92)	0.916
$53,701-85,500	0.07	(-1.11, 1.24)	0.913	0.08	(-0.63, 0.78)	0.832
$85,501-163,300	0.05	(-1.07, 1.16)	0.934	-0.48	(-1.15, 0.19)	0.161
Parent status[4]	-1.17	(-1.9, -0.44)	0.002	-0.58	(-1.02, -0.14)	0.009
Perceived illness[1]	2.55	(1.58, 3.52)	<.0001	0.93	(0.35, 1.51)	0.002
Time taken off for illness[1]	0.47	(-0.95, 1.88)	0.521	0.61	(-0.24, 1.47)	0.159
Relationship strain[1]	3.87	(3.25, 4.48)	<.0001	1.99	(1.62, 2.36)	<0.001
Survey timeframe (Reference, April)						
May	-0.26	(-0.98, 0.46)	0.480	-0.42	(-0.9, 0.06)	0.087
June	0.24	(-0.47, 0.94)	0.507	-0.41	(-0.85, 0.03)	0.065
COVID-19 Caseload[1,5]	-0.04	(-0.46, 0.37)	0.421	0.443	(-0.10, 0.16)	0.623
Intercept	31.99	(30.02, 33.97)	<.0001	-0.07	(-0.09, -0.06)	<0.001

1 Repeated measures evaluated at each survey timepoint
2 Categorization consistent with 2020 census. Categories consolidated if reported by <5% of respondents.
3 Defined as respiratory therapist or patient care technician
4 Defined as having one or more child for whom the participant is a guardian
5 Proportion of COVID-19 cases per maximal bed capacity at the identified hospital of employment by the participants

Values are from linear mixed models with fixed effects accounting for time, POS, and all variables of interest that were significant in the univariate analysis, and with random effects accounting for the hospital and participant.

Abbreviations: CI, confidence intervals.

ANXIETY

Higher POS was associated with lower anxiety (Table 3). Reporting perceived illness and endorsing circumstantial strain on personal relationships were associated with higher anxiety. Attending and trainee physician status were associated with lower anxiety. Having dependents and male sex were also associated with lower anxiety. The association between POS and anxiety was consistently observed across subgroups, but the effect was significantly moderated by occupation, relationship strain, and income. The magnitude of the inverse relationship was strongest among respiratory therapists and patient care technicians compared with other providers, those with relationship strain compared to those without, and those in lower compared to the highest income bracket (eTable 3). Higher POS remained associated with lower anxiety in sensitivity analysis (eTable 4).

MEDIATION ANALYSIS

Analysis of data from the first survey administration demonstrated that higher POS was associated with lower anxiety (Figure 2). Higher anxiety was associated with higher risk for burnout. Without adjusting for anxiety there was a significant direct effect of POS on burnout, such that higher POS was associated with lower risk for burnout. There was a negative indirect effect of POS on burnout through anxiety. The total of indirect and direct effects in the mediation analysis indicated that anxiety is a partial mediator of the association between POS and burnout. Mediation analysis of data from second and third survey administrations were similar (Figure 2; eTable 5-7).

Figure 2. Anxiety mediates the association between Perceived Organizational Support and Burnout.

Discussion

Burnout is characterized by emotional exhaustion, depersonalization, and loss of self-worth.[21] Up to 50% of doctors and nurses report symptoms of burnout,[22-24] with health care organizations cited as a leading cause.[25-30] In this longitudinal prospective survey-based study of healthcare workers during the COVID-19 pandemic, higher POS was associated with lower anxiety and risk for burnout. Anxiety was found to partially mediate the association between POS and burnout, suggesting that in the context of crisis, strategies aimed at alleviating anxiety might reduce burnout.

Burnout among healthcare providers is a prevalent and well-documented phenomenon[31] and has been associated with adverse clinical outcomes,[32,33] reduced productivity,[34] and increased rate of medical errors.[4] Aside from the consequences to providers and their patients, burnout represents a serious financial burden; $4.6 billion lost annually is attributed to physician attrition and loss of clinical hours secondary to burnout.[5] Heavy workloads, changing clinical roles, reduced decision latitude (i.e., the ability to exercise control over work-related responsibilities), and lack of support from supervisors have been cited as causes of increased occupational stress,[35] a key predictor of burnout.[36] During this pandemic additional stressors such as lack of personal protective equipment and fear of contagion have been shown to augment the risk for burnout.[1,31] These issues have threatened the stability of our healthcare organizations and, therefore, the COVID-19 medical crisis has also functionally become an *organizational crisis.*[37] As a result, health-related performance and quality outcomes may, in part, depend on how a healthcare organization manages to allocate resources and human capital[38] and whether it effectively develops policies to protect and support its employees. In our study, we observed that higher POS reduced risk for burnout. We speculate that this inverse relationship may reflect consequences that result from how organizations behave in response to periods of crisis. The *threat-rigidity effect* postulates that organizations may centralize power and reduce communication in periods of crisis,[38] which may, in turn, lead to reduced POS if the employee feels that his/her/their employer is

not concerned about the employee's well-being.[39] By the same token, as our data would indicate, if employees perceive support from their organizations, risk of burnout may decrease.

While previous studies have demonstrated that providers frequently cite organizational causes of burnout;[26-31] characteristics of the person may also contribute to burnout during periods of crisis.[40] We identified several individual factors that were associated with risk for burnout. Modifiable factors included strain on personal relationships. Our findings are consistent with data from others demonstrating that restrictions brought on by COVID-19 affect relationship quality and have consequences for mental health.[41] Other individual factors such as occupation and household income may also influence burnout.[31] Our finding that relative to respiratory therapists and patient care technicians, being a staff nurse is associated with increased risk of burnout reveals that provider-type may contribute to differences in pandemic-related burnout. We speculate that the degree to which individual factors influence burnout may vary depending on social support. The protective effect of having children that we and others have observed[42] and the aforementioned detrimental effect of COVID-19 related circumstances on personal relationships substantiate this theory. With respect to non-modifiable risk factors, the influence of gender on burnout in general remains unclear. In our study, male gender just reached the threshold of significance on primary analysis and was significantly associated with reduced burnout risk in the sensitivity analysis. Some studies have observed that the prevalence of burnout is higher in women,[43] while others have shown that women have decreased risk of burnout.[44] These conflicting findings may indicate that confounders obfuscate whether gender is independently associated with burnout. It may be that women are at higher risk for certain facets, such as emotional exhaustion, whereas men are predisposed to others, such as depersonalization.[45] Furthermore, there is evidence that gender disparities in mental health may be exacerbated by the pandemic and that disproportionate distribution of domestic responsibilities may contribute.[46] Our observation that individual factors contribute to burnout risk echoes others' work showing that effective

interventions may be tailored based on high-risk characteristics, personalized based on occupation, and/or focus on strengthening social support systems.[47]

Although various psychological conditions have been found to contribute to burnout, the effect of anxiety, especially in acute periods of threat, is relatively understudied.[48] Anxiety has both stable (i.e., trait) and dynamic (i.e., state) components. Trait anxiety describes the degree to which an individual may be prone to anxiety.[49] State anxiety, on the other hand, is a reaction to circumstances that are perceived as threatening,[50] such as the COVID-19 pandemic.[51] Our finding that anxiety partially mediates the effect of POS on burnout has been alluded to by others, who have theorized that work situations may trigger heightened anxiety and lead to burnout.[52] Additionally, existing literature is consistent with our observation that individual factors such as gender, occupation, and income may influence response to work-related stress and increase vulnerability to anxiety.[53] Understanding these relationships is the first step in developing interventions that address the multifaceted nature of burnout.

There are several limitations worth addressing. This study, although surveying individuals from multiple hospitals, is from a single healthcare system and geographical area which may limit external validity. In this study, attrition rates increased at later survey administrations, presumably due to survey fatigue. Therefore, missingness may not be at random, forcing respondent exclusion from the primary analysis. Further, the racial/ethnic homogeneity of our sample likely compromised our ability to accurately assess how sociodemographic factors influence burnout and anxiety in the context of a multifaceted construct such as POS. As this study was executed during the early months of the pandemic, variability in diagnostic testing across the healthcare system may have contributed to underreporting of hospital-specific rates of COVID-19. Finally, survey response was adequately powered to evaluate the *a priori* hypotheses; however, exploratory moderation analyses were underpowered.

Conclusion

During a health crisis, increasing the organizational support perceived by healthcare employees may reduce the risk for burnout through a reduction in anxiety. Improving the relationship between healthcare organizations and the individuals they employ may reduce detrimental effects of psychological distress among healthcare providers and ultimately improve patient care.

Article Information

Conflict of Interest Disclosures

No authors report disclosures, conflict of interest or relevant financial interests related to the content of the manuscript.

Funding/Support

Dr. Reitz was supported in part by the National Heart, Lung, and Blood Institute (5T32HL0098036) and National Institutes of Health (L30AG064730). These funding sources had no role in the design and conduct of the study; collection, management, analysis, and interpretation of the data; preparation, review, or approval of the manuscript; and decision to submit for publication.

Role of Funder/Sponsor:

The funders had no role in the design and conduct of the study; collection, management, analysis, and interpretation of the data; preparation, review, or approval of the manuscript; and decision to submit the manuscript for publication.

Acknowledgements

We would like to acknowledge Brenda Diergaarde, PhD Associate Professor, Human Genetics for sharing her expertise in the creation and use of the RedCap database. We would also like to thank Drs. Robert Handzel, Kristina Nicholson, Ariel Shensa, and Kenneth Lee for their support throughout the early phases of project development.

Author contributions

Drs. Reitz, Rosengart, and Myers take full responsibility for the integrity of the data and the accuracy of the data analysis.

Study concept and design: Reitz, Myers, Rosengart

Acquisition of data: Reitz, Myers, Rosengart, Campwala, Owoc

Interpretation of data: All authors

Analysis of data: Smith, Terhorst

Drafting of the manuscript: Reitz, Myers

Critical revision of the manuscript for important intellectual content: All authors

Study supervision: Myers, Rosengart

References

[1] Lai J, Ma S, Wang Y, et al. Factors associated with mental health outcomes among health care workers exposed to Coronavirus Disease 2019. *JAMA Netw Open.* 2020; 3(3):e203976. doi: 10.1001/jamanetworkopen.2020.3976.

[2] Shanafelt TD, Balch CM, Bechamps G, et al. Burnout and medical errors among American surgeons. *Ann Surg.* 2010; 251(6):995-1000. doi: 10.1097/SLA.0b013e3181bfdab3.

[3] Han S, Shanafelt TD, Sinsky CA, et al. Estimating the attributable cost of physician burnout in the United States. *Ann Intern Med.* 2019; 170(11): 784-790. doi: 10.7326/M18-1422.

[4] Melamed S, Shirom A, Toker S, Berliner S, Shapira I. Burnout and risk of cardiovascular disease: evidence, possible causal paths, and promising research directions. *Psychol Bull.* 2006; 132(3):327-53.

[5] Sun W, Fu J, Chang Y, Wang L. Epidemiological study on risk factors for anxiety disorder among Chinese doctors. *Journal of Occupational Health.* 2012; 54(1):1-8. doi: 10.1037/0033-2909.132.3.327.

[6] Muse LA, Stamper CL. Perceived organizational support: Evidence for a mediated associated with work performance. *Journal of Managerial Issues.* 2007; 19(4):517-535.

[7] Halbesleben JR, Rathert C. Linking physician burnout and patient outcomes: exploring the dyadic relationship between physicians and patients. *Health Care Manage Rec*. 2008; 33:29-39. doi: 10.1097/01.HMR.0000304493.87898.72.

[8] Perreault WD Jr. Controlling Order-Effect Bias. *The Public Opinion Quarterly.* 1976; 39(4): 544-551. doi: 10.1086/268251

[9] COVID-19 updates on testing, treatment, and visitor information. UPMC latest updates on COVID-19 website. Updated February 15, 2021. Accessed March 7, 2021. https://www.upmc.com/coronavirus.

[10] Rhoades L, Eisenberger R. Perceived organizational support: A review of the literature. *Journal of Applied Psychology.* 2002; 87: 698-714. doi: 10.1037/0021-9010.87.4.698.

[11] Eisenberger R, Huntington R, Hutchison S, Sowa D. Perceived organizational support. *Journal of Applied Psychology.* 1986; 71: 500-507. doi: 10.1037/0021-9010.71.3.500

[12] Professional Quality of Life Measure. ProQOL.org. Updated 2019. Accessed April 1, 2020. https://proqol.org/ProQol_Test.html

[13] Stamm BH. Professional Quality of Life Scale (PROQOL) Compassion satisfaction and compassion fatigue (PROQOL) Version 5. Updated 2009. Accessed April 1, 2020. https://www.proqol.org/uploads/ProQOL_5_English_Self-Score_3-2012.pdf

[14] Shensa A, Sidani JE, Dew MA, Escobar-Viera CG, Primack BA. Social media use and depression and anxiety symptoms: A Cluster Analysis. *Am J Health Behav.* 2018; 42(2):116-128. doi: 10.5993/AJHB.42.2.11.

[15] Worley JA. *A factor analytic study to evaluate the structure of the survey of perceived organizational support.* [PhD thesis]. Oklahoma State University; 2006.

[16] Geoffrion S, Lamothe J, Morizot J, Giguere C. Construct validity of the Professional Quality of Life (ProQol) scale in a sample of child protection workers. *Journal of Traumatic Stress.* 2019; 32(4): 566-576. doi: 10.1002/jts.22410.

[17] Teresi JA, Ocepek-Welikson K, Kleinman M, Ramirex M, Kim G. Measurement equivalence of the Patient Reported Outcomes Measurement Information System (PROMIS) Anxiety short forms in ethnically diverse groups. *Psychol Test Assess Model.* 2016; 58(1):182-219. doi: 10.1037/t03589-000

[18] Little RA, Rubin DB. *Statistical Analysis with Missing Data* (3rd ed). New Jersey: John Wiley and Sons, Inc; 2020.

[19] Singer JD, Willett JB. *Applied Longitudinal Data Analysis: Modeling Change and Event Outcomes*. New York: Oxford University Press; 2003.

[20] Hayes AF. Partial, conditional, and moderated mediation: Quantification, inference, and interpretation. *Communication Monographs.* 2018; 85: 4-40. doi: 10.1080/03637751.2017.1352100.

[21] STROBE Statement. Strobe-statement organization website. Updated 2009. Accessed March 7, 2021. https://www.strobe-statement.org/index.php?id=available-checklists.

[22] Okunogbe A, Meredith LS, Chang ET, Simon A, Stockdale SE, Rubenstein L V. Care coordination and provider stress in primary care management of high-risk patients. *J Gen Intern Med*. 2018;33(1):65-71. doi: 10.1007/s11606-017-4186-8.

[23] Kane L. Medscape National Physician Burnout & Suicide Report 2020: The Generational Divide. Medscape. Published 2020. https://www.medscape.com/slideshow/2020-lifestyle-burnout-6012460#14

[24] Van Mol MMC, Kompanje EJO, Benoit DD, Bakker J, Nijkamp MD, Seedat S. The prevalence of compassion fatigue and burnout among healthcare professionals in intensive care units: A systematic review. *PLoS One*. 2015;10(8):1-22. doi: 10.1371/journal.pone.0136955.

[25] Shanafelt TD, Gorringe G, Menaker R, et al. Impact of organizational leadership on physician burnout and satisfaction. *Mayo Clin Proc*. 2015;90(4):432-440. doi: 10.1016/j.mayocp.2016.10.004.

[26] Brindley PG, Olusanya S, Wong A, Crowe L, Hawryluck L. Psychological 'burnout' in healthcare professionals: Updating our understanding, and not making it worse. *J Intensive Care Soc*. 2019;20(4):358-362. doi: 10.1177/1751143719842794.

[27] Gates R, Workman A, Collier B. Job satisfaction and workplace stressors among surgical providers at a single institution. *J Am Acad Physician Assist*. 2019;32(11):42-47. doi: 10.1097/01.JAA.0000586332.19619.b6.

[28] Labrague LJ, McEnroe-Petitte DM, Leocadio MC, Van Bogaert P, Cummings GG. Stress and ways of coping among nurse managers: An integrative review. *J Clin Nurs*. 2018;27(7-8):1346-1359. doi: 10.1111/jocn.14165.

[29] Rosenstein AH. Hospital administration response to physician stress and burnout. *Hosp Pract (1995)*. 2019;47(5):217-220. doi: 10.1080/21548331.2019.1688596.

[30] Sehlen S, Vordermark D, Schäfer C, et al. Job stress and job satisfaction of physicians, radiographers, nurses and physicists working in radiotherapy: A multicenter analysis by the DEGRO Quality of Life Work Group. *Radiat Oncol*. 2009;4:1-9. doi: 10.1186/1748-717X-4-6.

[31] Stuijfzand S, Deforges C, Sandoz V, et al. Psychological impact of an epidemic/ pandemic on the mental health of healthcare professionals: a rapid review Suzannah. *BMC Public Health*. 2020;20(1):1230. doi: 10.1186/s12889-020-09322-z.

[32] Shanafelt T, Boone S, Tan L, et al. Burnout and Satisfaction with Work-Life Balance Among US Physicians Relative to The General US Population. *Arch Intern Med.* 2012; 172(18): 1377-85. doi: 10.1001/archinternmed.2012.3199.

[33] Panagioti M, Geraghty K, Johnson J, et al. Association between physician burnout and patient safety, professionalism, and patient satisfaction: a systematic- review and meta-analysis. *JAMA Intern Med*. 2018; 178:1317-1330. doi: 10.1001/jamainternmed.2018.3713.

[34] Wel A, Meier LL, Manser T. Emotional exhaustion and workload predict clinician-rated and objected patient safety. *Front Psychol*. 2014:5:1573. doi: 10.3389/fpsyg.2014.01573.

[35] Dewa CS, Loong D, Bonato S, Thanh NX, Jacobs P. How does burnout affect physician productivity? A systematic literature review. *BMC Health Serv Res*. 2014; 14:325. doi: 10.1186/1472-6963-14-325.

[36] Cocker F, Joss N. Compassion fatigue among healthcare, emergency and community service workers: A systematic review. *Int J Environ Res Public Health*. 2016;13(6):1-18. doi: 10.3390/ijerph13060618.

[37] van der Wal RAB, Wallage J, Bucx MJL. Occupational stress, burnout and personality in anesthesiologists. *Curr Opin Anaesthesiol*. 2018 Jun;31(3):351-356. doi: 10.1097/ACO.0000000000000587.

[38] Mishra AK. *Organizational responses to crisis: centrality of trust*. Appears in : Kramer, Roderick M. and Thomas Tyler (eds.) Trust In Organizations. Newbury Park, CA; Sage: 1996. 261-287.

[39] Staw B, Sandelands L, Dutton J. Threat-rigidity effects in organizational behavior: A multilevel analysis. *Administrative Science Quarterly*. 1981; 26: 501-524. doi: 10.2307/2392337.

[40] Moorman C, Zaltman G, Deshpande R. Relationships between providers and users of market research: The dynamics of trust within and between organizations. *Journal of Marketing Research*. 1992; 29: 314-328. doi: 10.2307/3172742.

[41] Kiesler S, Sproull L. Managerial response to changing environments: Perspectives on problem sensing from social cognition. *Administrative Science Quarterly*. 1982; 27: 548-570. doi: 10.2307/2392530.

[42] Pieh C, O Rourke T, Sudimir S, Probst T. Relationship quality and mental health during COVID-19 lockdown. *PLoS One.* 2020; 15(9):e0238906. doi: 10.1371/journal.pone.0238906.

[43] Chuang CH, Tseng P, Lin C, Lin K, Chen Y. Burnout in the intensive care unit professionals. *Medicine (Baltimore).* 2016; 95(50):e5629. doi: 10.1097/MD.0000000000005629.

[44] Embriaco N, Azoulay E, Barrau K, et al. High level of burnout in intensivists: prevalence and associated factors. *Am J Respir Crit Care Med.* 2007 Apr 1; 175(7):686-92. doi: 10.1164/rccm.200608-1184OC.

[45] Merlani P, Verdon M, Businger A, et al. Burnout in ICU caregivers: a multicenter study of factors associated to centers. *Am J Respir Crit Care Med.* 2011 Nov 15; 184(10):1140-6. doi: 10.1164/rccm.201101-0068OC.

[46] Raggio B, Malacarne P. Burnout in intensive care unit. *Minerva Anestesiol.* 2007 Apr; 73(4):195-200. doi: 10.1186/1471-2253-13-38.

[47] Almeida M, Shrestha AD, Stojanac D, Liller LJ. The impact of the COVID-19 pandemic on women's mental health. *Arch Womens Ment Health*. 2020; 23(6):741-748. doi: 10.1007/s00737-020-01092-2.

[48] Schaufeli W, Dirk Enzmann. *The burnout companion to study and practice: A critical analysis*. CRC press; 1998.

[49] Koutsimani P, Montgomery A, Georganta K. The relationship between burnout, depression, and anxiety: A systematic review and meta-analysis. *Front Psychol*. 2019; 10(284): 1-19. doi: 10.3389/fpsyg.2019.00284.

[50] Spielberger CD. *Theory and research on anxiety.* New York: Academic Press; 1966.

[51] Choi EPH, Hui BPH, Wan EYF. Depression and anxiety in Hong Kong during COVID-19. *Int J Environ Res Public Health*. 2020; 17 (10): 3740. doi: 10.3390/ijerph17103740.

[52] Turnipseed DL. Anxiety and burnout in the health care work environment. *Psychol Reports*. 1998; 82: 627–642. doi: 10.2466/pr0.1998.82.2.627.

[53] Vagg PR, Speilberger CD, Wasala CF. Effects of organizational level and gender on stress in the workplace. *International Journal of Stress Management.* 2002; 9: 243-261. doi: 10.1023/A:101996433134

Figure 1 Click here to access/download;Figure;Figure 1_PLoS.docx

Figure 1. Cohort accrual

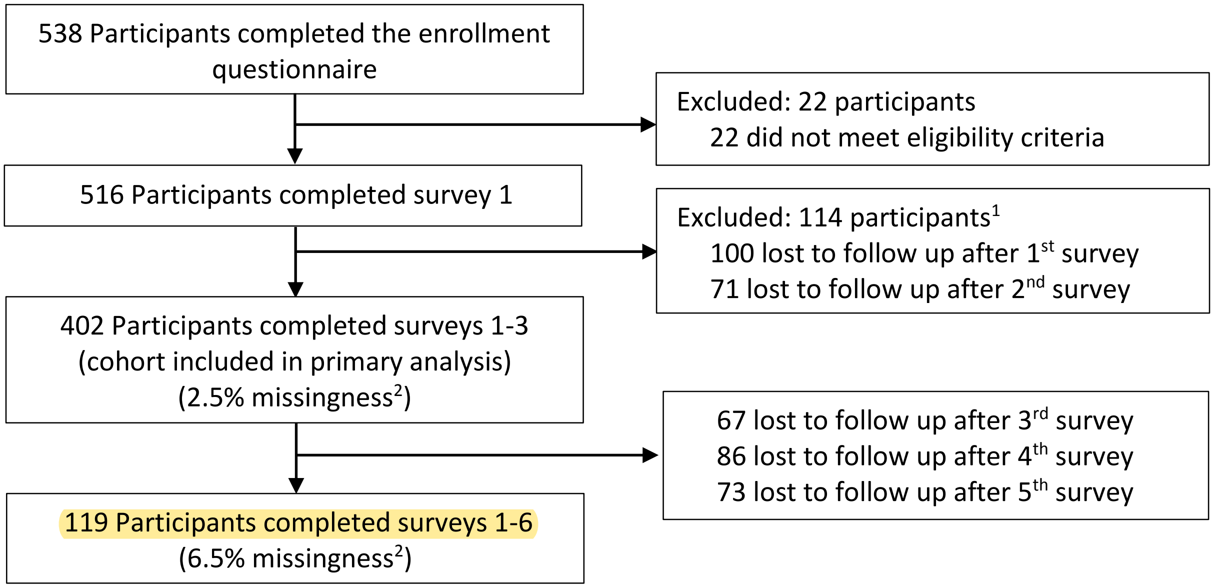

[1] Discrepancy between number excluded from primary analysis and number lost to follow-up stems from participants failing to complete consecutive surveys. As data from participants who completed two of the three initial surveys was included in the primary analysis, some individuals may have cursorily been considered lost to follow up after survey 1 but been included in the analysis if they returned to complete survey 3.

[2] Within survey missingness.

Figure 2. Anxiety mediates the association between perceived organizational support and burnout

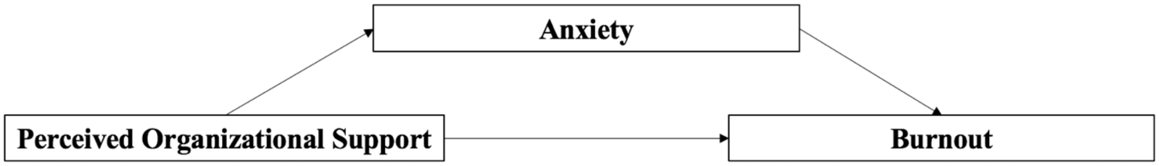

	Direct effect			Indirect effect	Total effect
**Study Month**[1]	Perceived organizational support on anxiety	Anxiety on risk of burnout	Perceived organizational support on risk of burnout	Perceived organizational support on risk of burnout	Perceived organizational support on risk of burnout (Anxiety as mediator)
	Effect size (95% CI)			*Effect size (95% CI)*	*Effect size (95% CI)*
April	-0.17 (-0.21, -0.12)	0.86 (0.71, 1.00)	-0.17 (-0.21, -0.13)	-0.06 (-0.09, -0.03)	-0.23 (-0.28, -0.19)
May	-0.16 (-0.20, -0.12)	0.77 (0.62, 0.91)	-0.16 (-0.20, -0.12)	-0.05 (-0.08, -0.03)	-0.21 (-0.26, -0.17)
June	-0.18 (-0.22, -0.14)	0.84 (0.68, 1.00)	-0.18 (-0.22, -0.14)	-0.07 (-0.10, -0.04)	-0.25 (-0.30, -0.20)

[1] Full adjusted models for each study month are available in Table S5-7 in the Supplementary Appendix.

Abbreviations: CI, confidence intervals.

Supplement

Click here to access/download

Supporting Information

Supplement_JOB.docx
