# Supplementary material for: Healthcare providers’ perceived support from their organization is associated with lower burnout and anxiety amid the COVID-19 pandemic
Source: PLoS One. 2021 Nov 19;16(11):e0259858. doi: 10.1371/journal.pone.0259858 (PMC8604356; doi:10.1371/journal.pone.0259858)
Supplement: Attachment — Submitted filename: PLOS_burnout_response_to_reviewers SPM 10_11.docx [file pone.0259858.s017.docx]

PLOS One Additional Editor Comments:

1. **Ethical considerations are missing from the methods section. PLS kindly add them.**

**Response:** The authors appreciate the request for transparency with regard to ethical considerations in study design and execution. We have revised the text of the methods section to specifically address the following: confidentiality and anonymity, informed consent, adverse events and benefits, and ability to withdraw from the study. Ethical considerations that were previously mentioned, including IRB approval and incentives, have been retained. The text of the recruitment script and informed consent document are present in the supplemental material. We feel that the manuscript now completely addresses ethical considerations.

**Related revised text:**

Methods: page 8, paragraph 1: “Participants were provided with study details, purpose of the investigation, and incentive information in the text of the recruitment e-mail and within the consent document. Participants were made aware that although the investigators did not anticipate that the study would cause adverse events, responding to survey items may evoke emotional responses such as stress. There were no direct benefits to the participant for completing surveys. Participants were free to withdraw from the study at any point; in this circumstance, all contact and data collection would cease and any existing data would be deleted upon request.”

2. **PLS clarify if respondents received anonymous questionnaires.**

**Response:** As requested above, we have clarified anonymization of the data. The participants were originally contacted via their email address. These email addresses were used to assign participants an alphanumeric code so that their serial data could be collected and stored in an anonymous fashion. One of the study investigators (IC) had access to the document that linked the email addresses to the anonymized participant code. To blind her to participant data, she did not have access to the stored data and was not involved with the statistical analysis.

**Related revised text:** Please see response to Editor’s comment # 1 above.

3. **Page 16, the highlighted lines are suitable for the introduction section.**

**Response:** We thank the editor for this suggestion. The authors believe that revising the manuscript to include the highlighted lines in the introduction rather than the discussion addresses comment #1 of the reviewer. Specifically, the introduction now clearly describes the importance of studying burnout and anxiety. We relate the significance of burnout to reduced productivity, adverse clinical outcomes, increased rate of medical errors, and the financial strain on the US economy. As per the request of the reviewer, we describe how existing literature has described the role of heavy workloads, changing clinical rolls, etc. on stress and burnout. The role of anxiety on burnout and during periods of crisis is also introduced. Finally, we have contextualized the influence of perceived organizational support on these constructs during the COVID-19 pandemic.

**Related revised text:** Introduction, Page 5, Paragraphs 2. “Burnout among healthcare providers is a prevalent and well-documented phenomenon^3^ and has been associated with adverse clinical outcomes,^4^ reduced productivity,^5^ and increased rate of medical errors.^6^ Aside from the consequences to providers and their patients, burnout represents a serious financial burden; $4.6 billion lost annually is attributed to physician attrition and loss of clinical hours secondary to burnout.^6^ Heavy workloads, changing clinical roles, reduced decision latitude (i.e., the ability to exercise control over work-related responsibilities), and lack of support from supervisors have been cited as causes of increased occupational stress,^7^ a key predictor of burnout.^8^ During this pandemic additional stressors such as lack of personal protective equipment and fear of contagion have been shown to augment the risk for burnout.^1,3^ These issues have threatened the stability of our healthcare organizations and, therefore, the COVID-19 medical crisis has also functionally become an *organizational crisis.*^9^ As a result, health-related performance and quality outcomes may, in part, depend on how a healthcare organization manages to allocate resources and human capital^10^ and whether it effectively develops policies to protect and support its employees.”

4. **The manuscript must be edited so that it follows scientific writing standards.**

**Response:** We have reviewed the submission guidelines on the PLOS ONE webpage. We have added line numbers to the manuscript file using continuous numbering. We have ensured that all abbreviations were defined upon first appearance in the text and that non-standard abbreviations were used only when they appeared three or more times in the test. We have consulted the Vancouver style guidelines for citing references to ensure that the format is correct. We have removed the reference to the following article, which has been retracted: Panagioti M, Geraghty K, Johnson J, et al. Association between physician burnout and patient safety, professionalism, and patient satisfaction: a systematic- review and meta-analysis. *JAMA Intern Med*. 2018; 178:1317-1330. doi: 10.1001/jamainternmed.2018.3713. The manuscript organization has been revised so that the acknowledgements and references appear in the acceptable order at the end of the manuscript. The title page has been revised according to the sample provided on the PLOS ONE webpage. Titles/professional credentials have been removed.

**Related revised text:** Please see title page, reference list, and ending comments.

**Reviewer comments:**

**Reviewer #1 (Remarks to the author): I appreciate the work to attend to the previous suggestions. Although most of the questions have been approached or answered appropriately, I consider that some of the points made in the previous review have not been addressed or, at least, not in depth. For this reason, I reiterate two of the recommendations made previously:**

**1. The introduction needs a theoretical justification of the study. Place special emphasis on clarifying why the analyzed variables are chosen, establishing where the work comes from and why it is important to address this. It may not have been analyzed how employer support affects burnout in workers during COVID-19, but what about other health crises or situations of high workload? Thus, I insist on the need to substantially improve the introduction. The discussion of the findings should be supported by a good initial introduction.**

**Response:** We apologize for not sufficiently addressing this in the initial revisions. The authors agree with the suggestion from this reviewer. We have revised the manuscript text to incorporate these details. Specifically, the introduction now clearly describes the importance of studying burnout and anxiety. We relate the significance of burnout to reduced productivity, adverse clinical outcomes, increased rate of medical errors, and the financial strain on the US economy. As per the request of the reviewer, we describe how existing literature has described the role of heavy workloads, changing clinical rolls, etc. on stress and burnout. The role of anxiety on burnout and during periods of crisis is also introduced. Finally, we have contextualized the influence of perceived organizational support on these constructs during the COVID-19 pandemic.

**Related revised text:** Introduction, Page 5, Paragraphs 1-2.

**2. Indicate the reliability of the instruments found in your study. It is understood that the reliability of the original scales is adequate, since otherwise they would not be valid instruments. However, what should be noted in the manuscript is the level of reliability of the scales for your sample.**

**Response:** We thank this reviewer for their comment. The internal consistency of the subscales using participants’ data have been calculated and added to the results section.

**Related revised text:** Results, Page 11, Paragraph 1: “Internal consistency calculated for subscales was as follows: SPOS=0.91, Burnout=0.85, and Anxiety= 0.89.”
